# Improving de novo sequence assembly using machine learning and comparative genomics for overlap correction

**DOI:** 10.1186/1471-2105-11-33

**Published:** 2010-01-15

**Authors:** Lance E Palmer, Mathaeus Dejori, Randall Bolanos, Daniel Fasulo

**Affiliations:** 1 Siemens Corporate Research, 755 College Road East, Princeton, NJ, USA; 2 Current Address: 454 Life Sciences, 15 Commercial Street Branford, CT 06405, USA

## Abstract

**Background:**

With the rapid expansion of DNA sequencing databases, it is now feasible to identify relevant information from prior sequencing projects and completed genomes and apply it to *de novo *sequencing of new organisms. As an example, this paper demonstrates how such extra information can be used to improve *de novo *assemblies by augmenting the overlapping step. Finding all pairs of overlapping reads is a key task in many genome assemblers, and to this end, highly efficient algorithms have been developed to find alignments in large collections of sequences. It is well known that due to repeated sequences, many aligned pairs of reads nevertheless do not overlap. But no overlapping algorithm to date takes a rigorous approach to separating aligned but non-overlapping read pairs from true overlaps.

**Results:**

We present an approach that extends the Minimus assembler by a data driven step to classify overlaps as true or false prior to contig construction. We trained several different classification models within the Weka framework using various statistics derived from overlaps of reads available from prior sequencing projects. These statistics included percent mismatch and *k*-mer frequencies within the overlaps as well as a comparative genomics score derived from mapping reads to multiple reference genomes. We show that in real whole-genome sequencing data from the *E. coli *and *S. aureus *genomes, by providing a curated set of overlaps to the contigging phase of the assembler, we nearly doubled the median contig length (N50) without sacrificing coverage of the genome or increasing the number of mis-assemblies.

**Conclusions:**

Machine learning methods that use comparative and non-comparative features to classify overlaps as true or false can be used to improve the quality of a sequence assembly.

## Background

*De novo *whole-genome shotgun sequencing requires three general steps: an initial sequencing step in which the target genome is redundantly sampled at random, producing reads via sequencing; assembly of the reads into a draft sequence; and finally, finishing and annotation of the genome. The second step in particular leads to numerous algorithmic challenges, and a number of approaches have been pioneered to deal with increasingly short reads and/or large target sequences as the capacity of sequencing facilities increases.

In general, two algorithmic approaches are currently employed for genome assembly. The first, which we call the overlap-contig-consensus (OCC) approach, is utilized in assemblers such as the Celera Assembler [1], Arachne [2], Atlas [3], and more recently CABOG [4] and Edena [5]. Implementations of OCC first calculate the overlaps between all pairs of reads, then use the overlap information to produce contigs, and finally generate the consensus sequence for the contigs. The second, which we call the de Bruijn approach, was first adopted from prior algorithmic work on sequencing by hybridization in the Euler-DB [6] assembler. This approach and related approaches have been used in assemblers such as Euler-SR [7], ALLPATHS [8], and Velvet [9]. The de Bruijn approach creates some form of "*k*-mer graph" from the reads and produces an assembly by transforming and traversing the graph. It is still unclear whether either of these paradigms is consistently superior to the other. But in general the success of the de Bruijn approach relies upon robust error detection in the reads to be practical, while the OCC approach requires fast and accurate overlap calculations. The latter problem is the focus of this work.

The OCC approach is best described by thinking of the target genome as a large interval, and the reads as being sub-intervals of the genome. This picture is oversimplified because it does not model the presence of errors and polymorphisms--particularly indels and structural polymorphisms--within the input reads, but is sufficient to introduce the general concept. Dealing with the presence of substantial polymorphisms in the context of OCC has been addressed in other work [10].

It is worth noting a few properties of the OCC approach. First, if the true overlaps between the reads were somehow known exactly, then contig formation on this ideal data set would be equivalent to Interval Graph Realization, a trivial problem. The implication is that overlapping is the more critical step. Second, overlapping reads should have a perfect--or nearly-perfect in the presence of sequencing errors--sequence alignment between their overlapping regions, and this observation is the fundamental basis for overlap detection algorithms. Finally, when overlap detection algorithms make errors, the most common and most damaging are false positives due to the presence of repeat sequences that create good alignments between reads even though those reads do not represent overlapping intervals on the genome. Incorporating such overlaps into contigs produces mis-assemblies.

Because comparing all pairs of reads for overlaps is a time-consuming process in large data sets, developers of overlapping methods have focused on efficient methods for finding alignments in large sequence collections. They have then generally equated the resulting alignments with the overlap relationships between the reads, despite the fact that the presence of an alignment between the ends of two reads is necessary but not sufficient evidence for an overlap. When this problem has been considered, it has been addressed with simple heuristics. For example, the Celera Assembler attempts to avoid repeat-induced overlaps by screening for known repeat sequences, and CABOG and the UMD Overlapper [11,12] attempt to reduce false positives with heuristic selection of "good" *k*-mers that are used to seed (CABOG) or confirm (UMD Overlapper) overlaps.

Since adequate solutions exist for fast alignment searching, for example the algorithm of Rasmussen [13], this work focuses specifically on answering the critical follow-up question: *given that an alignment exists between two reads, do they really overlap? *In contrast to prior work, this question is approached as a formal classification problem. Alignment features are defined and classifiers such as C4.5 decision tree, Naive Bayes and Random Forest are trained and employed within the Weka framework [14] (a Java-based machine learning library) to label overlaps as either true or false. We show that by culling out the false overlaps and thus providing an overlap set that is closer to ideal, we are able to achieve longer contigs that cover a larger percentage of the genome. Finally, yet perhaps most importantly, since large-scale sequencing and finished genomes are now ubiquitous, we show how we can leverage previous, related sequencing projects, if available, in the *de novo *assembly pipeline. While, other comparative assemblers, such as AMOScmp [14] map the reads to a single reference genome, we show how multiple reference genome sequences can be used to help assemble genomes.

Rather than produce a whole new genome assembler, we demonstrate our method by introducing it as a module into Minimus [15], which is part of the Modular Open-source Assembler (AMOS) project. Although Minimus is not as sophisticated as some other assemblers, AMOS is notable for being well-engineered and specifically designed to accept external modules and is thus an ideal test vehicle.

## Results and Discussion

### Evaluation of 454 Reads from E. coli

#### Overlap statistics

To determine if classification of overlaps can improve the assembly of sequencing reads, 454 (GS20) sequencing reads from *Salmonella enterica *serovar *Typhi *strain E983139 were used to build training models within the Weka framework, and 454 (GS FLX) reads from *Escherichia coli *strain K12 substrain MG1655 were used as a test set to assemble. The *Salmonella *reads represented an approximately 8× coverage of the genome, while the *E. coli *reads represented an 18× coverage. Reads from both strains were mapped to their respective genomes to determine the ground truth in regards to whether any predicted overlaps were true or false overlaps. Because the completed genome for strain E983139 was not available, the *S. enterica *serovar *Typhi *Ty2 genome, which shares approximately 96% identity with the E983139 reads (data not shown), was used instead.

**Figure 1:**
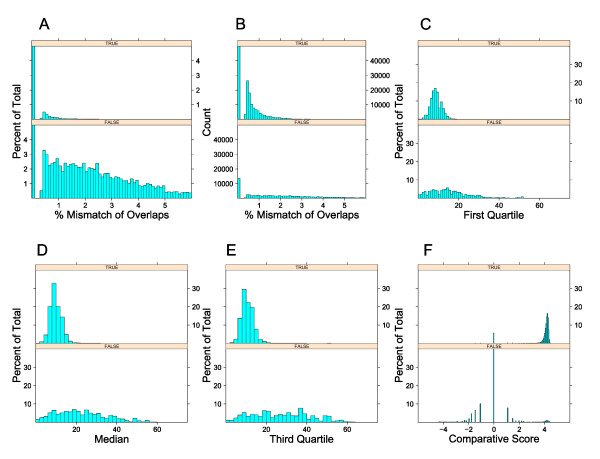
**Overlap statistics for E. coli MG1655 reads**. The percent mismatch of the alignment between the two reads (a, b), the first quartile *k*-mer frequency of *k*-mers within the overlap (c), the median *k*-mer frequency (d), the third quartile *k*-mer frequency (e), and the comparative overlap score (f) are plotted for both true and false overlaps. The results are normalized for percentages of total overlaps for each of the true and false overlaps (a, c, d, e, f) or by overall count (b). The number of total true overlaps with 0 mismatches is 5,209,686.

The AMOS hash-overlap [11] program was used to identify potential overlaps. Various statistics for these overlaps were calculated as described in the methods. These statistics included the percent mismatch within the overlap, the first, second, and third quartile *k*-mer frequencies, and a comparative genomics score. The quartile frequencies were derived by taking each *k*-mer within an overlap and calculating a normalized frequency of the *k*-mer within all reads. These frequencies were sorted from low to high, and the value of the frequency at each quartile of the distribution of frequencies was calculated. The distributions of these statistics as a function of the percentage of overlaps were calculated for these features within *E. coli *MG1655(Figure 1) to show that in fact these features could partially discriminate true from false overlaps. As a percentage of total overlaps within the true or false categories, the false overlaps had a larger tail for the percent mismatch score (Figure 1a). Since there were a large number of true overlaps compared to false overlaps (5,320,945 and 57,317 respectively), the total number of true overlaps with having 1% to 2% mismatches was still higher than false overlaps (Figure 1b). The *k*-mer distributions are shown in Figure 1c-e. As expected, false overlaps tended to have a greater *k*-mer frequency. To see if reads from false overlaps tended to be in repetitive parts of the genome, BLAT [16], with a minimum score (matches-mismatches) of 50, was used to determine the number of times each read mapped to the reference genome. Reads from true overlaps mapped on average 1.15 times to the reference genome, while reads from false overlaps mapped on average 4.59 to the reference genome. Finally a comparative genomics score was generated by mapping the reads to a set of related *E. coli *genomes (Figure 1F). A positive score was generated when the top match of each read mapped to the same location within a genome. A negative score was generated when the top match of each read was not in the same location within a genome. See Methods for a detailed description of the comparative genomics score.

#### Classification accuracy

As true and false overlaps had different distributions for the features that were tested, we next wanted to see if these features could be used to train classifiers that could be used to predict true and false overlaps. The accuracy of various machine learning algorithms in distinguishing true and false overlaps was explored. For the results shown in Table 1, both comparative and non-comparative features were used. *Salmonella *reads were used to generate the training models used below, and *E. coli *reads were used as the test set. For the comparative score, genomes with less than 91% identity to *Salmonella *Ty2 or *E. coli *MG1655 were used. Accuracy was defined as the number of correctly predicted overlaps (both true and false), divided by the total number of overlaps. The false positive rate was defined as the number of overlaps incorrectly predicted as positive, divided by the total number of actual false overlaps. The false negative rate was defined as the number of overlaps incorrectly predicted as false divided by the total number of actual true overlaps. The J48 classifier had the highest accuracy (99.28%) and the second lowest false positive rate (37.7%). The Naïve Bayes classifier with default parameters produced the lowest false postive rate (12.2%) but had the highest false negative rate of the machine learning classifiers (2.04% compared to 0.183% with the J48 classifier). Using kernel estimation with Naïve Bayes improved the accuracy of the classifier and had the lowest false negative rate. Confusion matrices for the classifiers are shown in Additional File 1, Tables S4 and S5. One example decision tree for the J48 classifier using both comparative and non-comparative data is shown in Additional File 1, Table S7.

**Table 1:** Overlap classification accuracy

*Classifier*	Accuracy^1^	False Pos. Rate^2^	False Neg. Rate^3^
J48	99.28%	37.7%	0.183%
NaïveBayes	97.82%	12.2%	2.04%
NaïveBayes (-K) ^4^	99.24%	44.5%	0.127%
Random Forest	99.20%	45.9%	0.149%
UMD Overlapper	74.90%	76.5%	23.3%

^1^Accuracy is defined as the number of true positive and true negative overlaps divided by the total number of overlaps

^2^The false positive rate is defined as the number of false positive overlaps divided by the number of false overlaps

^3^The false negative rate is defined as the number of false negative overlaps divided by the number of true overlaps

^4^NaïveBayes with kernel estimation

We wanted to compare the accuracy of our classifier with other methods of curating overlaps, which are typically rule-based. The latest version of the UMD Overlapper program [12] uses a simple rule involving *k*-mer frequencies to label overlaps as reliable and unreliable. Although this classification is not intended to be utilized for labeling overlaps as strictly true or false, it is similar in spirit, and we evaluated how well the reliable and unreliable categories aligned to true and false overlaps respectively (See Additional File 1, Table S6). The overall accuracy of the reliability labeling was only 74.90%. The false positive rate was 76.5% and the false negative rate was 23.7%.

#### Assembly of E. coli sequencing reads

**Figure 2:**
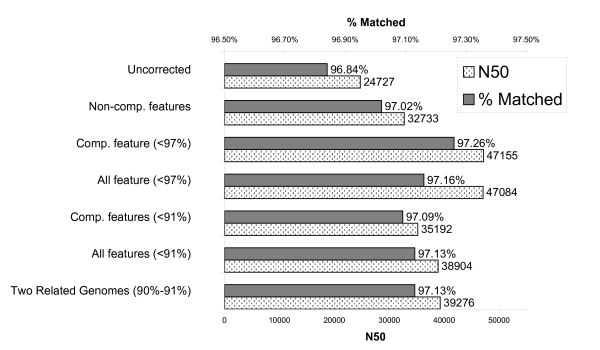
**Assembly of E. coli strain MG1655**. Statistics from overlaps derived from *S. typhi *training reads were used to train a J48 Weka model. Overlaps from the MG1655 test data were classified based on this model and any overlaps predicted to be false were removed. The remaining overlaps were used in the assembly of MG1655. The N50 contig length of the final assembly as well as the percentage of the reference MG1655 genome matched by the contigs are plotted. Within parenthesis, the percent cutoff for strains to be analyzed with the comparative score is shown. For the 'One related genome' and 'Two related genomes' data, only one (ATCC8739 for the test set) and two (ATCC8739 and E24377A for the test set) related genomes, respectively, for the training and test sets were used.

The features from the *S. typhi *training set were used to generate several training models using classifiers such as J48, Naïve Bayes and Random Forest within the Weka framework. Overlaps from the *E. coli *reads were generated and classified using the training models. After removal of overlaps predicted to be false, the reads were assembled using Minimus. The N50 score and the percentage of the reference genome matched by contigs were calculated for each assembly (Figure 2). The N50 score was defined as the maximum contig size where all contigs of that size and larger covered 50% of the genome. A higher N50 score generally represents the presence of larger contigs and thus a better assembly of the reads. As a control, we first assembled *E. coli *reads without classification and removal of overlaps predicted to be false. This assembly had an N50 score of 24,727 and 96.84% of the reference genome was matched.

While several classifiers were tested, including NaïveBayes and Random Forest, the J48 classifier generally, but not always, produced better assembly results based on N50 scores and the percentage of the reference genome matched. We also attempted to use support vector machines (SVM) within Weka, but the classifier failed to finish due to the large number of overlaps involved. The results presented here represent the values from the J48 classification. The first set of training models were built using the non-comparative features such as mismatch percentage and the *k*-mer frequencies. The J48 model produced an N50 size 32,733 and a percent genome matched of 97.02%. The next model to be analyzed was built using comparative features alone. For generating the comparative score within the test set, we excluded those strain that were highly similar (>97% identity) to the test strain (See Additional File 1, Table S1 for list of strains and percent identity). By using this compartive score alone, the assembly produced contigs with an N50 score of 47,155 and a percent match to the reference genome of 97.26%. Assembling the reads using both the comparative and non-comparative features yielded an assembly with an N50 score of 47,084 and a percent match to the reference genome of 97.16%. If we reduced the allowable strains for the comparative feature to those less than 91% identity, the N50 score and percent match for the comparative features alone dropped to 35,182 and 97.09%. Using both comparative and non-comparative features increased these values to 38,904 and 97.13%, respectively. In addition to using the N50 statistic, we examined the N statistic for a range of N with several of the assemblies (Additional File 1, Figure S1). Again, the assembly using both comparative (using genomes with less than <91% identity) and non-comparative features provided the best results for most of the values of N between 1 and 99. Additional File 1, Figure S2 shows the range of N statistics for assemblies using genomes <97% identical.

For some genome sequencing projects, there may not be a large number of related genomes, as there are for *E. coli *and *Salmonella*. Therefore, the assembly was repeated using only two related genomes for the training and test set. For *Salmonella *Ty2, *S. enterica *serovar *Paratyphi *strain ATCC9150 (91.6% identity to *Salmonella *Ty2) and *S. enterica *serovar *Typhimurium *LT2 (89.6% identity to Ty2 and 87.9% identity to ATCC9150) were used as related genomes. For the MG1655 test assembly, *E. coli *strains ATCC8739 (92.6% identity to MG1655) and E24377A (92.1% identity to MG1655 and 90.0% identity to ATCC8739) were used as related genomes. For the J48 classifier the N50 score was 40,597 and the percentage of the reference genome matched by the contigs was 97.13%. If only Ty2 for *Salmonella *and ATCC8739 for *E. coli *were used as related genomes, the N50 score for J48 decreased to 35,164 and the percentage of the reference genome matched fell to 3.06. These results suggest that there does not need to be a large number of related genomes required for comparisons. See Additional File 1, Figure S3 for a graph of these values.

#### Analysis of assembly quality

**Figure 3:**
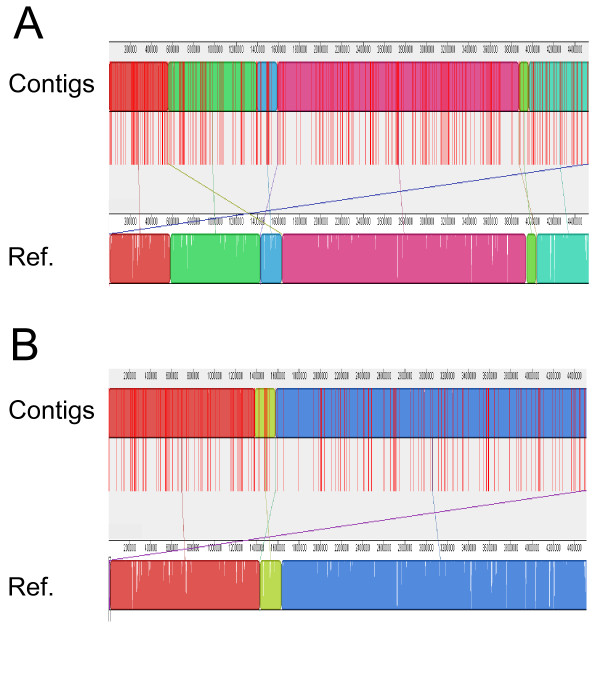
**Visualizing alignments of contigs**. Contigs of assembled *E. coli *MG1655 reads from the uncorrected (top) and J48 corrected (bottom) overlaps were mapped to the MG1655 genome using SNAPPER and ordered in respect to their position. MAUVE was used to visualize alignments of assembled contigs to the reference genome. Each segment represents a matching alignment between the contigs and reference genome. There may be more than one segment per contig. Red vertical lines represent contig boundaries. Colored blocks represent regions where contigs are in the correct order. Colored lines connect corresponding blocks between the reference genome and the assembled contigs. White spaces within the blocks within the reference genome indicate regions not represented within a contig.

**Figure 4:**
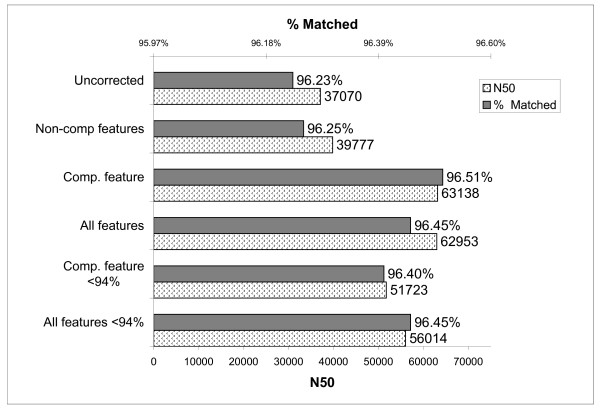
**Assembly of S. aureus strain JH1**. Statistics from overlaps derived from the JH9 training reads were used to train a J48 Weka model. Overlaps from the JH1 test data were classified based on this model and any overlaps predicted to be false were removed. The remaining overlaps were used in assembly of JH1. The N50 contig length of the final assembly as well as the percentage of the reference JH1 genome matched by the contigs are plotted. Within parenthesis, the percent cutoff for strains to be analyzed with the comparative score is shown.

DNADIFF was used to identify gross mis-assemblies of the *E. coli *sequencing reads [17]. We focused on the assembly using non-comparative features combined with the comparative feature using strains less than 97% identity. DNADIFF reported two potential misjoins and a single tandem deletion for both the uncorrected and corrected assemblies. As a qualitative view of the assemblies, we used MAUVE [18] to align assembled contigs that had been ordered by SNAPPER [19] to the finished reference genome. Figure 3 shows the alignment of the uncorrected (Figure 3). Figure 4a shows the uncorrected assembly, while 3b shows the assembly after overlaps predicted to be false by the J48 classifier were removed. For both 3a and 3b, the assembled contigs are shown mapped to the reference sequence. Contig boundaries are shown by vertical red bars. Different colored blocks represent regions where contigs are in the correct order. Breaks in the correct order of the contigs with respect to the reference genome are shown by the boundaries between colored block, and by the crossing colored lines. While there are several cases of contigs not being in the correct order (shown by the colored lines crossing), manual inspection of the breaks show that these occur in repetitive regions and do not represent true errors, only differences between alignment algorithms of SNAPPER and MAUVE. We were not able to detect any gross mis-assemblies from the alignments.

### Evaluation of Sanger Reads from S. aureus

To determine if classification of overlaps could be applied to other types of data sets, we next analyzed Sanger reads from *Staphylococcus aureus*. Reads from strain JH9 were used as a training set and reads from strain JH1 were used in the test set. Both sets of reads represented an approximately 9× coverage of their respective genomes. The assembly of JH1 using uncorrected overlaps produced contigs with an N50 score of 37,070 that matched 96.23% of the reference genome (Figure 4). Using non-comparative features produced little improvement (39,777 N50 score and 96.25% coverage of the reference genome). With comparative feature using all *S. aureus *strains except JH1 and JH9, the assembly was improved (63,138 N50 score and 96.51% coverage), while including non-comparative features reduced the assembly quality slightly (62,953 N50 score and 96.45% coverage). However when only strains with less than 94% identity to JH1 and JH9 were used for the comparative score, the results of using both non-comparative and comparative features were better than comparative features alone (51,723 N50 and 96.4% coverage for comparative features alone vs 56,014 N50 score and 96.45% coverage for both comparative and non-comparative features). The a plot of the N statistic for range of N is shown in Additional File 1, Figure S3.

## Conclusions

By providing a more accurate approximation of the true overlap structure of the input reads, overlap classification can simplify the task of contig construction and thereby create superior assemblies. In particular, the addition of information from related genomes strengthens the quality of the assembly, without sacrificing the flexibility of the *de novo *framework for a purely comparative assembly process with a single reference genome such as provided by AMOScmp [14]. Use of related genomes can potentially be generalized to other aspects of *de novo *assembly, including the de Bruijn approach where this information can be used to resolve pairs of branches. From the results shown here, if highly similar genomes are available, a purely comparative approach will likely yield better results. However, if these sequences are not available, using a machine learning approach using non-comparative and comparative features from more distantly related reference genomes can improve sequence assembly.

The overlap classification implementation described here was tested in a rather simple genome assembler, and may produce a smaller percentage improvement of the N50 length if placed in more sophisticated assemblers. On the other hand, the bacterial genomes utilized as test cases are not particularly repetitive; it is hypothesized that the results could be even stronger in repeat-rich genomes.

In our experiments, the size of the training set did not have an impact on the results (assuming basic sufficiency) (data not shown), allowing the use of smaller training sets than test sets. Once trained, the decision tree can be quickly applied to new overlaps, and thus the computational time taken by the overlap correction module was in line with other modules in the assembly process. The alignment statistics should be computed during the overlap process to maximize efficiency and the reads must be searched for high-quality hits to reference genome(s), but neither of these presents an excessive computational burden compared to other phases of genome assembly. It should therefore be possible to apply overlap correction to larger genomes.

## Methods

The importance of an accurate set of overlapping reads for the quality of subsequent sequence assembly methods has been examined by Roberts *et al. *[11,12] by showing a significant improvement of both error rate and coverage. In this paper we propose a supervised classification framework to discriminate between true and false overlaps, using machine learning techniques. In what follows, we will define our approaches and describe the employed features.

A summary of our complete overlap correction pipeline is shown in Additional File 1, Figure S5.

### DNA Sequences

Sequencing reads (GS FLX) from *Escherichia coli *strain K12 substrain MG1655 were downloaded from the NCBI short read archive (SRA000156) [20], while sequencing reads for *Salmonella typhi *strain E98319 were retrieved from the European Read Archive (ERA000001) [21,22]. Sequencing reads from *Staphylococcus aureus *strains JH1 and JH9 were downloaded from the NCBI trace archive. The *S. aureus *reads were sequenced by standard Sanger sequencing, while the *E. coli *and *S. typhi *reads were sequenced with the Roche/454 GS FLX sequencer. Reference genome sequences for all available *E. coli*, *S. typhi*, and *S. aureus *genomes were obtained from NCBI Genbank (See Additional File 1, Tables S1, S2 and S3 for lists of all sequences used and their accession numbers). Paired end data for the sequencing reads was not used. Sequencing reads were quality trimmed and vector trimmed using the CABOG Gatekeeper program [4]. Reads were mapped to their corresponding reference genome using SNAPPER [19], and the coordinates were used to calculate the ground truth in terms of whether the overlaps generated by the hash overlapper were true or false overlaps. The reads were converted to the AMOS bank format for assembly.

### Generating Overlap Features

The AMOS hash-overlap program with a minimum overlap length of 30 bases and a maximum read disagreement rate of 6% was used to calculate overlap candidates. A number of statistics were then calculated from the alignment of each overlap. Basic overlap statistics included the percent identity of the overlap, defined as the number of matches over the length of the overlap region; the length of the overlap; the number of mismatches; and the number of gaps of any length. An additional set of *k*-mer statistics were calculated by first counting the frequency of all *k*-mers in the input set of reads (*k *= 17 for *E. coli *and 16 for *S. aureus *in our tests). These included the first, second and third quartile frequencies of each *k*-mer from each read within the overlap region, relative to the expected sequence coverage; the percentage of *k*-mers above the threshold (1.5*mean frequency), which we call *overrepresented k-mers*, relative to the total number of *k*-mers in the overlap region; and the number of *k*-mers with a frequency of exactly 1 (these are more likely to be sequencing errors than true disagreements in the reads). For the results shown in this study, only the number of mismatches/overlap length and the quartile *k*-mer frequencies were used for non-comparative features.

For each overlap, a comparative genomics score was also generated. Reads were mapped using SNAPPER to strains similar to the genome being trained or tested (See Additional File 1, Tables S1, S2, and S3). For the *Salmonella typhi *training set, all strains with the exception of the reference genome Ty2 were used. For all other sets, strains with less than the percentage specified in the results were used for training or testing. When executing SNAPPER, the mersize parameter was set to 16, minmatchidentity was set to 80, and minmatchcoverage was set to 80. For each genome to be compared, each pair of overlapping reads were mapped to the genome sequence. All possible combinations of locations were compared, and the maximum overlapping and maximum non-overlapping scores were calculated. For example, if Reads A and B overlapped and each mapped twice to a reference genome at positions X and Y, there would be two overlapping combinations (A at X, B at X and A at Y, B at Y) and two non-overlapping combinations (A at X, B at Y and A at Y, B at X). For each combination of reads, the total number of bases matching each of the reference genomes represented the score. For the entire set of genomes that the overlapping reads were mapped to, the top overlapping and top non-overlapping scores were determined. The final comparative score was calculated as log_2_(|max *s*_*ovl *_- max *s*_*non-ovl *_+ 1|), if max *s*_*ovl *_> max *s*_*non*-*ovl *_and log_2_(max *s*_*non*-*ovl *_- max *s*_-*ovl *_+ 1) otherwise where max *s*_*ovl *_denotes the top overlapping score and max *s*_*non*-*ovl *_the top non-overlapping score respectively. The final score was 0 if the top overlapping score was equal to the top non-overlapping score. The score was undefined if one or both of the reads did not map.

### Overlap Classification

Let **P **= {*p*_1_, *p*_2_, ..., *p*_*N*_} be a set of read pairs, where *N *denotes the number of read pairs and each read pair *p*_*i *_is represented by a feature vector *x*_*i*_. A training set *S *is defined as **S **= {(*x*_1_, *y*_1_), (*x*_2_, *y*_2_), ..., (*x*_*N*_, *y*_*N*_)} where *y*_*i *_is a binary value representing the true label of the *i*th read pair. In the learning procedure a classification function *C*: **x **→ *y*(**x**) maps the input feature vector **x **to the output feature vector *y*(**x**). Instead of formulating a suitable mathematical function *C *explicitly as in the UMD Overlapper [11,12], we used a machine learning approach to learn this function based on existing data. We attempted to use four different machine learning algorithms within the Weka framework (a Java-based machine learning library) [23]. These classifiers included support vector machines, J48, Random Forest and NaïveBayes. A classifier based on support vector machines failed due to memory issues with the large number of features. Two types of classifiers based on decision trees (DT) were used. These were the J48 implementation of C4.5 [24] and the Random Forest classifier, which uses multiple DT. A DT is best described by an acyclic graph in which the interior nodes specify testing of a single attribute of a feature vector and the nodes indicate the class of the decision. The tree structure is learned by recursively splitting the sample set, with each subset giving rise to one new vertex connected with an edge to its parent. This procedure continues until all samples at each leaf belong to the same class. The working flow of DT is similar to a logical tree structure that starts from the topmost node, and every decision of the node determines the direction of next node movement until the end of the tree branch node is reached. The last classifier used was the NaïveBayes (NB) classifier. The NB classifier uses a probabilistic model for classification. The classifier assumes that all features for classification are independent. The NB classifier was used with default values, which assumes a normal distribution of values, and with the -K value which uses a kernel density estimator and does not assume a normal distribution.

For training and testing the classifiers, the overlap statistics for each training set were calculated and the dataset generated was utilized to train classifiers. Subsequently for each test set, the overlapping reads and their statistics were generated as described above. The trained model was then plugged into the Minimus pipeline and used to classify each overlap as **true **or **false**. Overlaps predicted to be **false **were removed from the AMOS bank and excluded from the subsequent assembly process.

### Performance Evaluation

The performance of the overlap correction approach was evaluated two-fold. First, it was assessed based on its predictive performance in terms of true positives (the number of overlapping read-pairs classified as overlaps), false positives (the number of non-overlapping read-pairs classified as overlaps) and false negatives (the number of overlapping read-pairs classified as non-overlapping). Secondly, performance was assessed based on the N50 length (the shortest contig size where all contig sizes greater than or equal to this length sum up to 50% of the reference genome length) from the final assembly and the percentage of matched sequence relative to the reference. The DNADIFF [17] program was used to determine the percentage of the published genome that is covered by the assembly and the number of mis-assemblies. For visualization of the assemblies, contigs were mapped to the reference genome using SNAPPER and ordered according to position. Contigs were concatenated with 10 'N's added between each. The combined sequence was written to a FASTA formatted file. MAUVE [18] was used to align the resulting sequence to the reference genome.

## Authors' contributions

All authors contributed to conceiving and developing the methods described in this paper. MD developed the learning pipeline. LEP performed the data analysis. All authors read and approved the final manuscript.

## Supplementary Material

Additional file 1 **Supplementary Information**. This file contains supplementary figures, list of strains, and confusion matrices for testing machine learning algorithms. Click here for file
