# Supplementary material for: Improving de novo sequence assembly using machine learning and comparative genomics for overlap correction
Source: BMC Bioinformatics. 2010 Jan 15;11:33. doi: 10.1186/1471-2105-11-33 (PMC2824677; doi:10.1186/1471-2105-11-33)
Supplement: Additional file 1 — Supplementary Information. This file contains supplementary figures, list of strains, and confusion matrices for testing machine learning algorithms. [file 1471-2105-11-33-S1.PDF]

Additional File 1
Supplementary Information

Improving *de novo* sequence assembly using machine learning and comparative genomics for overlap correction

Lance E. Palmer, Mathaeus Dejori, Randall Bolanos and Daniel Fasulo

Supplementary Figure S1

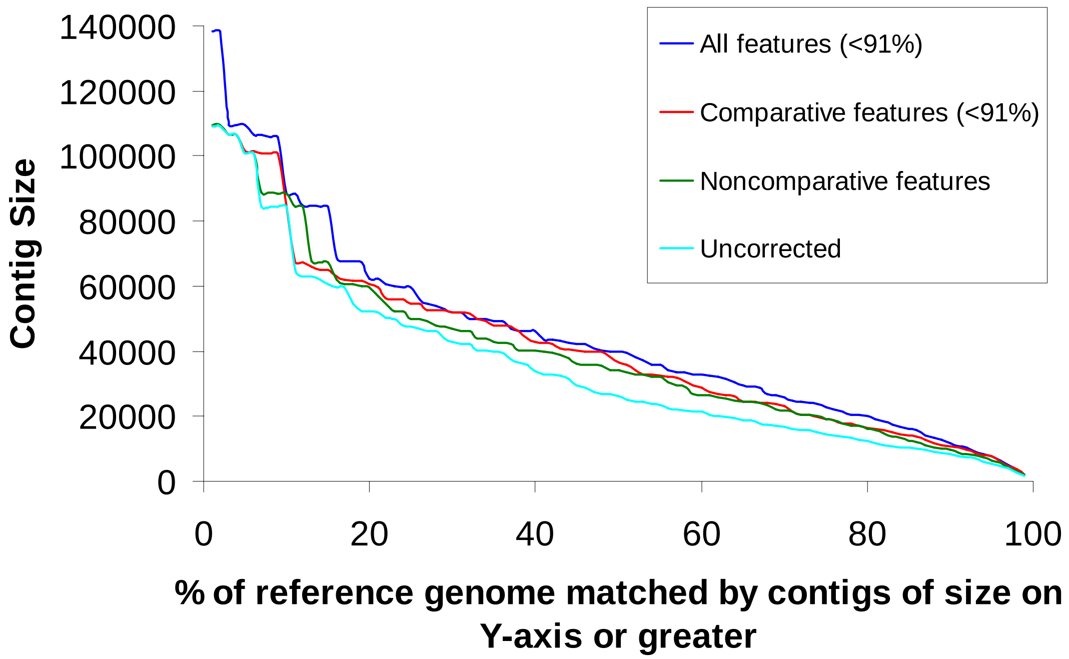

N statistic plot for assembled contigs of *E. coli*
Assembled contigs from *E. coli* MG1655 reads were ordered by size. The smallest contig size such that all contigs of that size or larger could cover N% of the genome where N ranged from 1 to 99 was determined and plotted with respect to N.

Supplementary Figure S2

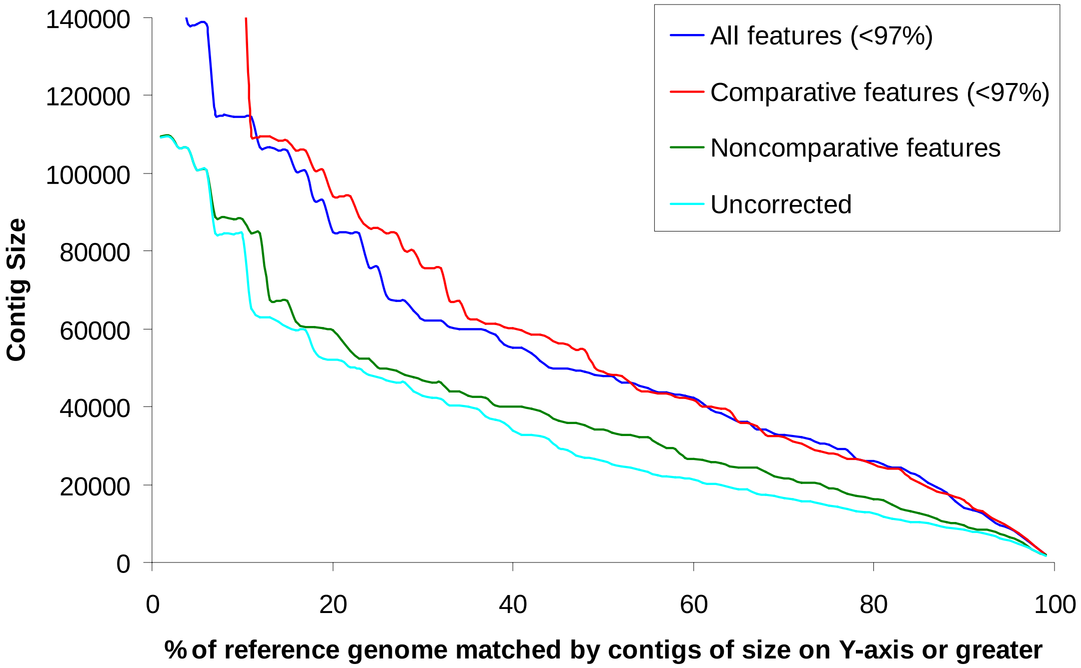

N statistic plot for assembled contigs of *E. coli*
Assembled contigs from *E. coli* MG1655 reads were ordered by size. The smallest contig size such that all contigs of that size or larger could cover N% of the genome where N ranged from 1 to 99 was determined and plotted with respect to N.

Supplementary Figure S3

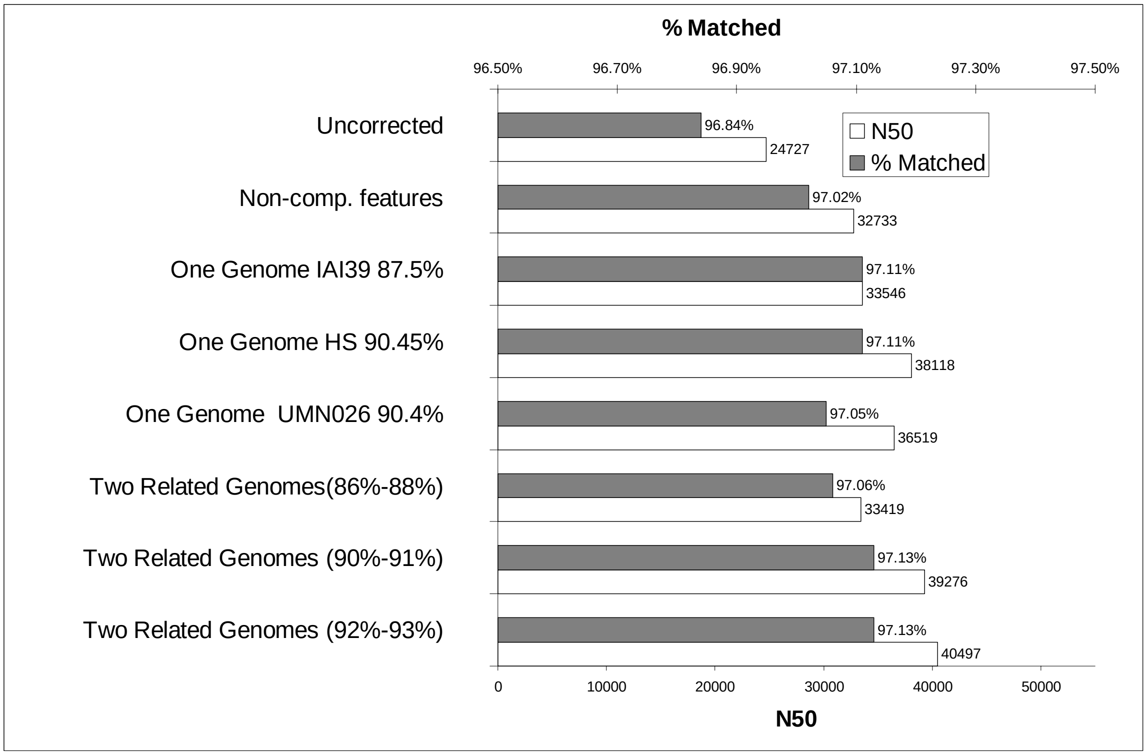

Assembly of *E. coli* strain MG1655 using a minimal set of related genomes. Statistics from overlaps derived from an *S. typhi* training reads were used to train a J48 Weka model. Overlaps from the MG1655 test data were classified based on this model and any overlaps predicted to be false were removed. The remaining overlaps were used inthe assembly of MG1655. The N50 contig length of the final assembly as well as the percentage of the reference MG1655 genome matched by the contigs are plotted. Uncorrected assemblies or assemblies with overlaps predicted to be false based on non-comparative features are shown. In addition, overlaps removed based on non-comparative or comparative features use one or two related genomes. For the training sets, *S. typhi* strains ATCC9150 and LT2 were used. For the test sets, different *E. coli* genomes were used. For the single related genome sets, the related strain is given. For the two related genome sets, there were three different test sets. For 86% to 88% range, strains S88 and IAI39 were used. For 90% to 91%, strains HS and UMN026 were used. For 92%-93%, strains 55989 and IAI1 were used.

Supplementary Figure S4

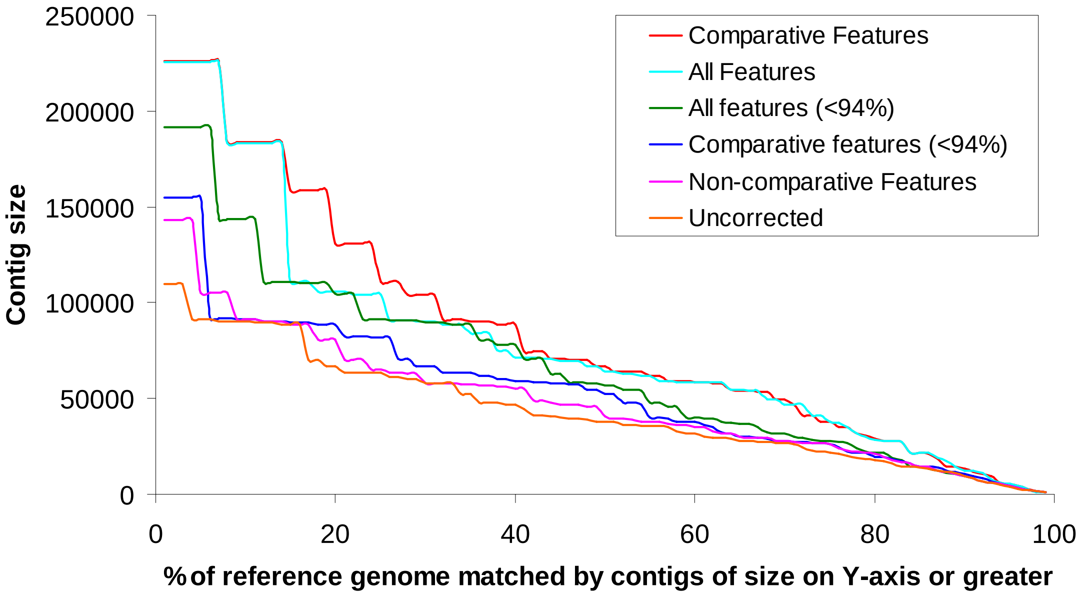

Cumulative N statistic for assembled contigs of *S. aureus*
Assembled contigs from *S. aureus* JH1 reads were ordered by size. The smallest contig size such that all contigs of that size or larger could cover N% of the genome where N ranged from 1 to 99 was determined and plotted with respect to N.

Supplementary Figure S5

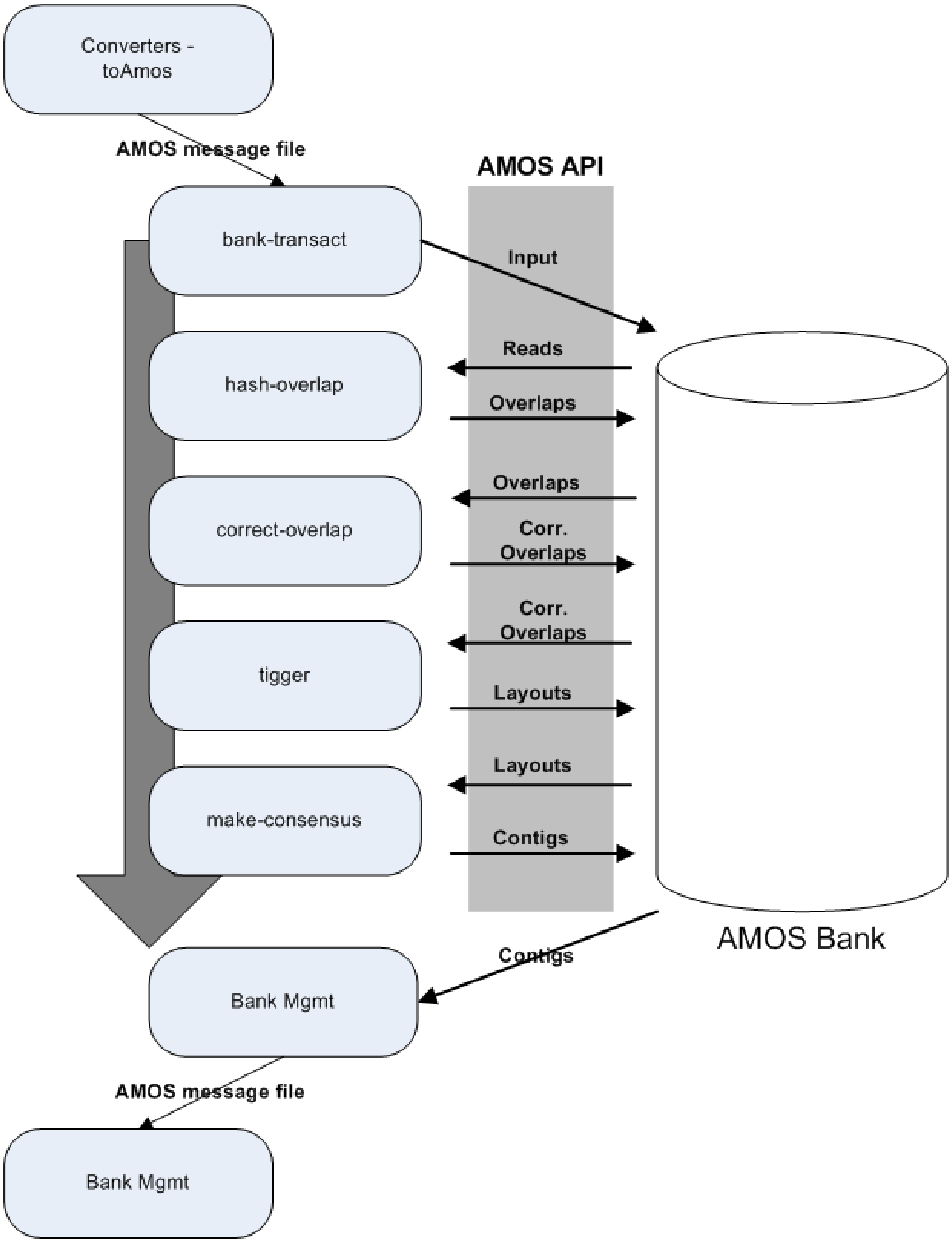

Overview of the overlap correction pipeline using the AMOS framework. The order of execution of the individual modules is shown by the arrow. Figure modified from Sommer et al. (2007).

Supplementary Table S1: *E. coli strains* used for comparative genomics

Strain	Accession	% Identity to MG1655
Escherichia_coli_K_12_substr__MG1655	NC_000913	100.00%
Escherichia_coli_W3110	AC_000091	99.85%
Escherichia_coli_K_12_substr__DH10B	NC_010473	97.36%
Escherichia_coli_C_ATCC_8739	NC_010468	92.59%
Escherichia_coli_E24377A	NC_009801	92.08%
Escherichia_coli_SE11	NC_011415	92.06%
Escherichia_coli_55989	NC_011748	91.60%
Escherichia_coli_IAI1	NC_011741	91.47%
Escherichia_coli_HS	NC_009800	90.45%
Escherichia_coli_UMN026	NC_011751	90.40%
Escherichia_coli_O157H7_EDL933	NC_002655	89.32%
Escherichia_coli_O157H7	NC_002695	89.18%
Escherichia_coli_O157_H7_EC4115	NC_011353	88.98%
Escherichia_coli_SMS_3_5	NC_010498	88.75%
Escherichia_coli_IAI39	NC_011750	87.50%
Escherichia_coli_S88	NC_011742	86.35%
Escherichia_coli_APEC_O1	NC_008563	85.86%
Escherichia_coli_UTI89	NC_007946	85.62%
Escherichia_coli_CFT073	NC_004431	85.11%
Escherichia_coli_LF82	NC_011993	85.05%
Escherichia_coli_536	NC_008253	84.75%
Escherichia_coli_ED1a	NC_011745	84.47%
Escherichia_coli_0127_H6_E2348_69	NC_011601	84.01%

Supplementary Table S2: *Salmonella* strains used for comparative genomics.

Strain	Accession	% Identity to Ty2
Salmonella_enterica_serovar_Typhi_Ty2	NC_004631	100.00%
Salmonella_enterica_serovar_Paratyphi_A_AKU_12601	NC_011147	91.71%
Salmonella_enterica_Paratypi_ATCC_9150	NC_006511	91.63%
Salmonella_typhimurium_LT2	NC_003197	89.59%
Salmonella_enterica_serovar_Dublin_CT_02021853	NC_011205	89.48%
Salmonella_enterica_serovar_Newport_SL254	NC_011080	89.28%
Salmonella_enterica_serovar_Paratyphi_B_SPB7	NC_010102	88.98%
Salmonella_enterica_serovar_Heidelberg_SL476	NC_011083	88.41%
Salmonella_enterica_serovar_Agona_SL483	NC_011149	88.15%
Salmonella_enterica_serovar_Enteritidis_P125109	NC_011294	88.15%
Salmonella_enterica_serovar_Schwarzengrund_CVM19633	NC_011094	88.13%
Salmonella_enterica_serovar_Gallinarum_287_91	NC_011274	87.62%
Salmonella_enterica_Choleraesuis	NC_006905	87.51%
Salmonella_enterica_arizonae_serovar_62_z4_z23__	NC_010067	75.41%

Supplementary Table S3: *Staphylococcus aureus* strains used for comparative genomics

S. aureus strain	Chromomosome Acc #	Percent Identity To JH1
Staphylococcus_aureus_JH1	NC_009632	100.00%
Staphylococcus_aureus_JH9	NC_009487	99.94%
Staphylococcus_aureus_Mu3	NC_009782	96.18%
Staphylococcus_aureus_Mu50	NC_002758	96.14%
Staphylococcus_aureus_N315	NC_002745	94.79%
Staphylococcus_aureus_Newman	NC_009641	92.70%
Staphylococcus_aureus_USA300_TCH1516	NC_010079	92.61%
Staphylococcus_aureus_USA300	NC_007793	92.55%
Staphylococcus_aureus_NCTC_8325	NC_007795	92.34%
Staphylococcus_aureus_MW2	NC_003923	91.62%
Staphylococcus_aureus_aureus_MRSA252	NC_002952	91.19%
Staphylococcus_aureus_aureus_MSSA476	NC_002953	91.18%
Staphylococcus_aureus_COL	NC_002951	91.14%
Staphylococcus_aureus_RF122	NC_007622	87.78%

Supplementary Table S4: Confusion matrices for various classifiers
Models using non-comparative and comparative features from *Salmonella* overlaps were used to train various machine learning algorithms. These models were tested on *E. coli* MG1655 overlaps. For the comparative score, genomes <91% identity were used.

A) J48

		Predicted	
		T	F
Actual	T	5292176	9689
	F	28769	47628

B) Naïve Bayes

		Predicted	
		T	F
Actual	T	5193741	108124
	F	9353	67044

C) Naïve Bayes with kernel estimation

		Predicted	
		T	F
Actual	T	5292176	6751
	F	34031	42366

D) Random Forest

		Predicted	
		T	F
Actual	T	5293979	7886
	F	35028	41369

Supplementary Table S5: Confusion matrices for the *E. coli* test sets

A) J48 classification without comparative data

		Predicted T	Predicted F
Actual	T	5300339	1526
	F	52502	23895

B) J48 classification with only comparative data (<97% identity)

		Predicted T	Predicted F
Actual	T	5296031	5834
	F	33442	42955

C) J48 classification with comparative (<97% identity) and non-comparative statistics

		Predicted T	Predicted F
Actual	T	5293408	8457
	F	21973	54424

D) J48 classification with only comparative data (<91% identity)

		Predicted T	Predicted F
Actual	T	5294296	7569
	F	53530	22867

E) J48 classification with comparative (<91% identity) and non-comparative statistics

		Predicted T	Predicted F
Actual	T	5292176	9689
	F	28769	47628

Supplementary Table S6: Confusion matrices for UMD overlapper. The UMD overlapper identifies overlaps as reliable or non-reliable. The following tables show how accurate this classification is.

A) UMD overlapper w/ E. coli comparative + non-comparative data

		Predicted	
		T	F
Actual	T	3944427	1200860
	F	134943	41354

Supplementary Table S7

J48 Decision Tree

```
comp_overlap <= 1.47
|   thirdQuartile <= 21
|   |   num_mismatches/overlap_length <= 0.014354
|   |   |   comp_overlap <= -0.9
|   |   |   |   num_mismatches/overlap_length <= 0.006601
|   |   |   |   |   secondQuartile <= 13: T (140.5/14.0)
|   |   |   |   |   secondQuartile > 13
|   |   |   |   |   |   comp_overlap <= -3.99: F (6.79/0.79)
|   |   |   |   |   |   comp_overlap > -3.99
|   |   |   |   |   |   |   secondQuartile <= 14: F (5.58/0.57)
|   |   |   |   |   |   |   secondQuartile > 14: T (14.81/3.0)
|   |   |   |   num_mismatches/overlap_length > 0.006601
|   |   |   |   |   thirdQuartile <= 10
|   |   |   |   |   |   comp_overlap <= -1.87: T (3.04/1.0)
|   |   |   |   |   |   comp_overlap > -1.87
|   |   |   |   |   |   |   comp_overlap <= -1.28: F (17.23/0.23)
|   |   |   |   |   |   |   comp_overlap > -1.28
|   |   |   |   |   |   |   |   firstQuartile <= 3
|   |   |   |   |   |   |   |   |   secondQuartile <= 3: F (2.02/0.02)
|   |   |   |   |   |   |   |   |   secondQuartile > 3: T (8.06/2.0)
|   |   |   |   |   |   |   |   firstQuartile > 3: F (13.24/1.24)
|   |   |   |   |   thirdQuartile > 10: T (29.84/8.0)
|   |   |   comp_overlap > -0.9
|   |   |   |   num_mismatches/overlap_length <= 0.004149: T (2378.64/74.04)
|   |   |   |   num_mismatches/overlap_length > 0.004149
|   |   |   |   |   thirdQuartile <= 19
|   |   |   |   |   |   thirdQuartile <= 8
|   |   |   |   |   |   |   secondQuartile <= 7
|   |   |   |   |   |   |   |   thirdQuartile <= 7: T (67.31/6.04)
|   |   |   |   |   |   |   |   thirdQuartile > 7
|   |   |   |   |   |   |   |   |   firstQuartile <= 3
|   |   |   |   |   |   |   |   |   |   num_mismatches/overlap_length <= 0.005495: F (4.06/0.06)
|   |   |   |   |   |   |   |   |   |   num_mismatches/overlap_length > 0.005495: T (11.23/2.0)
|   |   |   |   |   |   |   |   |   firstQuartile > 3: T (26.13/3.0)
|   |   |   |   |   |   |   secondQuartile > 7
|   |   |   |   |   |   |   |   firstQuartile <= 4: F (7.14/0.14)
|   |   |   |   |   |   |   |   firstQuartile > 4: T (19.62/7.0)
|   |   |   |   |   |   thirdQuartile > 8
|   |   |   |   |   |   |   num_mismatches/overlap_length <= 0.010309: T (440.94/16.0)
|   |   |   |   |   |   |   num_mismatches/overlap_length > 0.010309
|   |   |   |   |   |   |   |   thirdQuartile <= 15: T (84.99/9.0)
|   |   |   |   |   |   |   |   thirdQuartile > 15
|   |   |   |   |   |   |   |   |   firstQuartile <= 7: F (11.12/2.12)
|   |   |   |   |   |   |   |   |   firstQuartile > 7
|   |   |   |   |   |   |   |   |   |   num_mismatches/overlap_length <= 0.01087
|   |   |   |   |   |   |   |   |   |   |   comp_overlap <= 0.55: F (5.24/0.24)
|   |   |   |   |   |   |   |   |   |   |   comp_overlap > 0.55: T (2.09)
|   |   |   |   |   |   |   |   |   |   num_mismatches/overlap_length > 0.01087: T (27.92/2.01)
|   |   |   |   |   thirdQuartile > 19
|   |   |   |   |   |   secondQuartile <= 18
|   |   |   |   |   |   |   firstQuartile <= 13
|   |   |   |   |   |   |   |   secondQuartile <= 15: F (3.16/1.16)
|   |   |   |   |   |   |   |   secondQuartile > 15
|   |   |   |   |   |   |   |   |   num_mismatches/overlap_length <= 0.009434: F (4.08/1.08)
|   |   |   |   |   |   |   |   |   num_mismatches/overlap_length > 0.009434: T (4.02)
|   |   |   |   |   |   |   firstQuartile > 13: F (8.34/0.34)
```

```
|   |   |   |   |   |   secondQuartile > 18
|   |   |   |   |   |   |   comp_overlap <= 0.84: T (23.08/1.0)
|   |   |   |   |   |   |   comp_overlap > 0.84
|   |   |   |   |   |   |   |   comp_overlap <= 1.2: F (2.1/0.1)
|   |   |   |   |   |   |   |   comp_overlap > 1.2: T (2.1)
|   |   num_mismatches/overlap_length > 0.014354
|   |   |   comp_overlap <= -0.55: F (402.02/26.78)
|   |   |   comp_overlap > -0.55
|   |   |   |   thirdQuartile <= 15
|   |   |   |   |   num_mismatches/overlap_length <= 0.037313: T (149.27/30.04)
|   |   |   |   |   num_mismatches/overlap_length > 0.037313
|   |   |   |   |   |   thirdQuartile <= 4: T (3.01)
|   |   |   |   |   |   thirdQuartile > 4
|   |   |   |   |   |   |   thirdQuartile <= 12: F (14.15/0.13)
|   |   |   |   |   |   |   thirdQuartile > 12
|   |   |   |   |   |   |   |   thirdQuartile <= 13: T (3.05/0.03)
|   |   |   |   |   |   |   |   thirdQuartile > 13: F (2.04/0.03)
|   |   |   |   thirdQuartile > 15
|   |   |   |   |   comp_overlap <= 1.24
|   |   |   |   |   |   firstQuartile <= 12
|   |   |   |   |   |   |   secondQuartile <= 18
|   |   |   |   |   |   |   |   comp_overlap <= 0.46: F (142.39/5.28)
|   |   |   |   |   |   |   |   comp_overlap > 0.46
|   |   |   |   |   |   |   |   |   thirdQuartile <= 18
|   |   |   |   |   |   |   |   |   |   num_mismatches/overlap_length <= 0.025478: T (6.03/0.0)
|   |   |   |   |   |   |   |   |   |   num_mismatches/overlap_length > 0.025478: F (2.03/0.01)
|   |   |   |   |   |   |   |   |   thirdQuartile > 18: F (29.04/0.03)
|   |   |   |   |   |   |   secondQuartile > 18
|   |   |   |   |   |   |   |   secondQuartile <= 19: F (9.08/1.08)
|   |   |   |   |   |   |   |   secondQuartile > 19: T (3.01)
|   |   |   |   |   |   firstQuartile > 12
|   |   |   |   |   |   |   thirdQuartile <= 19
|   |   |   |   |   |   |   |   comp_overlap <= 0
|   |   |   |   |   |   |   |   |   firstQuartile <= 13: T (10.08/1.0)
|   |   |   |   |   |   |   |   |   firstQuartile > 13
|   |   |   |   |   |   |   |   |   |   firstQuartile <= 14: F (6.06/1.06)
|   |   |   |   |   |   |   |   |   |   firstQuartile > 14: T (20.15/4.0)
|   |   |   |   |   |   |   |   comp_overlap > 0: T (10.08/0.0)
|   |   |   |   |   |   |   thirdQuartile > 19
|   |   |   |   |   |   |   |   secondQuartile <= 19: F (26.08/0.08)
|   |   |   |   |   |   |   |   secondQuartile > 19
|   |   |   |   |   |   |   |   |   thirdQuartile <= 20: T (3.05)
|   |   |   |   |   |   |   |   |   thirdQuartile > 20: F (3.02/0.02)
|   |   |   |   |   comp_overlap > 1.24: T (15.06/1.01)
|   thirdQuartile > 21
|   |   num_mismatches/overlap_length <= 0.004237
|   |   |   firstQuartile <= 32
|   |   |   |   comp_overlap <= 0
|   |   |   |   |   firstQuartile <= 27
|   |   |   |   |   |   thirdQuartile <= 25
|   |   |   |   |   |   |   comp_overlap <= -2
|   |   |   |   |   |   |   |   secondQuartile <= 21
|   |   |   |   |   |   |   |   |   thirdQuartile <= 24
|   |   |   |   |   |   |   |   |   |   comp_overlap <= -3.73: F (3.16/0.16)
|   |   |   |   |   |   |   |   |   |   comp_overlap > -3.73: T (5.27/1.0)
|   |   |   |   |   |   |   |   |   thirdQuartile > 24: T (2.01)
|   |   |   |   |   |   |   |   secondQuartile > 21: F (23.22/0.22)
|   |   |   |   |   |   |   comp_overlap > -2
|   |   |   |   |   |   |   |   comp_overlap <= -0.46
|   |   |   |   |   |   |   |   |   thirdQuartile <= 24: T (46.93)
|   |   |   |   |   |   |   |   |   thirdQuartile > 24: F (3.04/0.04)
|   |   |   |   |   |   |   |   comp_overlap > -0.46
```

```
|   |   |   |   |   |   |   |   |   firstQuartile <= 12
|   |   |   |   |   |   |   |   |   |   secondQuartile <= 23
|   |   |   |   |   |   |   |   |   |   |   secondQuartile <= 15: F (7.15/0.15)
|   |   |   |   |   |   |   |   |   |   |   secondQuartile > 15: T (19.37/1.0)
|   |   |   |   |   |   |   |   |   |   secondQuartile > 23: F (19.01/0.01)
|   |   |   |   |   |   |   |   |   firstQuartile > 12
|   |   |   |   |   |   |   |   |   |   firstQuartile <= 18: T (51.54)
|   |   |   |   |   |   |   |   |   |   firstQuartile > 18
|   |   |   |   |   |   |   |   |   |   |   secondQuartile <= 23: F (24.68/6.68)
|   |   |   |   |   |   |   |   |   |   |   secondQuartile > 23: T (85.29/30.0)
|   |   |   |   |   |   thirdQuartile > 25
|   |   |   |   |   |   |   thirdQuartile <= 50
|   |   |   |   |   |   |   |   firstQuartile <= 20
|   |   |   |   |   |   |   |   |   comp_overlap <= -0.9
|   |   |   |   |   |   |   |   |   |   firstQuartile <= 8: T (3.11/0.0)
|   |   |   |   |   |   |   |   |   |   firstQuartile > 8: F (51.72/1.67)
|   |   |   |   |   |   |   |   |   comp_overlap > -0.9
|   |   |   |   |   |   |   |   |   |   secondQuartile <= 35
|   |   |   |   |   |   |   |   |   |   |   thirdQuartile <= 26
|   |   |   |   |   |   |   |   |   |   |   |   secondQuartile <= 21
|   |   |   |   |   |   |   |   |   |   |   |   |   secondQuartile <= 13: F (4.02/0.02)
|   |   |   |   |   |   |   |   |   |   |   |   |   secondQuartile > 13: T (2.1)
|   |   |   |   |   |   |   |   |   |   |   |   secondQuartile > 21: F (16.16/0.16)
|   |   |   |   |   |   |   |   |   |   |   thirdQuartile > 26
|   |   |   |   |   |   |   |   |   |   |   |   thirdQuartile <= 35
|   |   |   |   |   |   |   |   |   |   |   |   |   firstQuartile <= 13
|   |   |   |   |   |   |   |   |   |   |   |   |   |   firstQuartile <= 10: T (8.21/1.0)
|   |   |   |   |   |   |   |   |   |   |   |   |   |   firstQuartile > 10: F (9.16/1.14)
|   |   |   |   |   |   |   |   |   |   |   |   |   firstQuartile > 13
|   |   |   |   |   |   |   |   |   |   |   |   |   |   thirdQuartile <= 27
|   |   |   |   |   |   |   |   |   |   |   |   |   |   |   firstQuartile <= 18: T (2.03)
|   |   |   |   |   |   |   |   |   |   |   |   |   |   |   firstQuartile > 18: F (2.01/0.01)
|   |   |   |   |   |   |   |   |   |   |   |   |   |   thirdQuartile > 27: T (46.02/1.02)
|   |   |   |   |   |   |   |   |   |   |   |   thirdQuartile > 35
|   |   |   |   |   |   |   |   |   |   |   |   |   thirdQuartile <= 39: F (12.06/2.04)
|   |   |   |   |   |   |   |   |   |   |   |   |   thirdQuartile > 39: T (9.14/2.04)
|   |   |   |   |   |   |   |   |   |   secondQuartile > 35: F (19.06/1.02)
|   |   |   |   |   |   |   |   firstQuartile > 20: F (351.47/29.29)
|   |   |   |   |   |   |   thirdQuartile > 50
|   |   |   |   |   |   |   |   comp_overlap <= -0.55: F (20.36/2.33)
|   |   |   |   |   |   |   |   comp_overlap > -0.55
|   |   |   |   |   |   |   |   |   secondQuartile <= 47
|   |   |   |   |   |   |   |   |   |   secondQuartile <= 42
|   |   |   |   |   |   |   |   |   |   |   thirdQuartile <= 55: F (11.03/2.01)
|   |   |   |   |   |   |   |   |   |   |   thirdQuartile > 55: T (11.86)
|   |   |   |   |   |   |   |   |   |   secondQuartile > 42: T (47.12/2.0)
|   |   |   |   |   |   |   |   |   secondQuartile > 47
|   |   |   |   |   |   |   |   |   |   firstQuartile <= 20: T (5.28/1.03)
|   |   |   |   |   |   |   |   |   |   firstQuartile > 20: F (6.15/0.08)
|   |   |   |   |   firstQuartile > 27
|   |   |   |   |   |   thirdQuartile <= 32
|   |   |   |   |   |   |   comp_overlap <= -2.72: F (7.09/0.09)
|   |   |   |   |   |   |   comp_overlap > -2.72: T (122.55)
|   |   |   |   |   |   thirdQuartile > 32
|   |   |   |   |   |   |   thirdQuartile <= 50: F (27.27/2.17)
|   |   |   |   |   |   |   thirdQuartile > 50
|   |   |   |   |   |   |   |   secondQuartile <= 49
|   |   |   |   |   |   |   |   |   secondQuartile <= 46
|   |   |   |   |   |   |   |   |   |   firstQuartile <= 29: T (9.0/1.0)
|   |   |   |   |   |   |   |   |   |   firstQuartile > 29: F (15.02/1.01)
|   |   |   |   |   |   |   |   |   secondQuartile > 46: T (10.0)
|   |   |   |   |   |   |   |   secondQuartile > 49: F (9.06/0.03)
```

```
|   |   |   |   comp_overlap > 0
|   |   |   |   |   num_mismatches/overlap_length <= 0.003802
|   |   |   |   |   |   firstQuartile <= 14
|   |   |   |   |   |   |   comp_overlap <= 1.24
|   |   |   |   |   |   |   |   thirdQuartile <= 31: T (14.18/1.0)
|   |   |   |   |   |   |   |   thirdQuartile > 31: F (5.12/1.11)
|   |   |   |   |   |   |   comp_overlap > 1.24
|   |   |   |   |   |   |   |   thirdQuartile <= 24
|   |   |   |   |   |   |   |   |   firstQuartile <= 12: T (2.05)
|   |   |   |   |   |   |   |   |   firstQuartile > 12: F (7.03/0.03)
|   |   |   |   |   |   |   |   thirdQuartile > 24: T (6.15/0.01)
|   |   |   |   |   |   firstQuartile > 14
|   |   |   |   |   |   |   firstQuartile <= 21: T (68.0/1.03)
|   |   |   |   |   |   |   firstQuartile > 21
|   |   |   |   |   |   |   |   comp_overlap <= 1.15
|   |   |   |   |   |   |   |   |   thirdQuartile <= 56
|   |   |   |   |   |   |   |   |   |   thirdQuartile <= 46
|   |   |   |   |   |   |   |   |   |   |   thirdQuartile <= 43
|   |   |   |   |   |   |   |   |   |   |   |   thirdQuartile <= 42: T (3.41/1.02)
|   |   |   |   |   |   |   |   |   |   |   |   thirdQuartile > 42: F (3.0/0.0)
|   |   |   |   |   |   |   |   |   |   |   thirdQuartile > 43: T (10.0/0.0)
|   |   |   |   |   |   |   |   |   |   thirdQuartile > 46: F (5.02/0.01)
|   |   |   |   |   |   |   |   |   thirdQuartile > 56: T (18.01/1.0)
|   |   |   |   |   |   |   |   comp_overlap > 1.15: T (16.18/0.01)
|   |   |   |   |   num_mismatches/overlap_length > 0.003802
|   |   |   |   |   |   thirdQuartile <= 32: T (2.09)
|   |   |   |   |   |   thirdQuartile > 32: F (13.02/1.02)
|   |   |   firstQuartile > 32: F (172.02/6.47)
|   |   num_mismatches/overlap_length > 0.004237
|   |   |   thirdQuartile <= 31
|   |   |   |   num_mismatches/overlap_length <= 0.014184
|   |   |   |   |   firstQuartile <= 27
|   |   |   |   |   |   num_mismatches/overlap_length <= 0.006969
|   |   |   |   |   |   |   firstQuartile <= 15
|   |   |   |   |   |   |   |   secondQuartile <= 25
|   |   |   |   |   |   |   |   |   comp_overlap <= -1.67: T (2.03)
|   |   |   |   |   |   |   |   |   comp_overlap > -1.67
|   |   |   |   |   |   |   |   |   |   firstQuartile <= 14
|   |   |   |   |   |   |   |   |   |   |   comp_overlap <= 0.46
|   |   |   |   |   |   |   |   |   |   |   |   thirdQuartile <= 25: T (4.04)
|   |   |   |   |   |   |   |   |   |   |   |   thirdQuartile > 25: F (2.13/0.13)
|   |   |   |   |   |   |   |   |   |   |   comp_overlap > 0.46
|   |   |   |   |   |   |   |   |   |   |   |   secondQuartile <= 18: F (3.03/0.03)
|   |   |   |   |   |   |   |   |   |   |   |   secondQuartile > 18: T (2.12)
|   |   |   |   |   |   |   |   |   |   firstQuartile > 14: F (12.08/0.08)
|   |   |   |   |   |   |   |   secondQuartile > 25: T (4.06)
|   |   |   |   |   |   |   firstQuartile > 15: T (20.2)
|   |   |   |   |   |   num_mismatches/overlap_length > 0.006969
|   |   |   |   |   |   |   thirdQuartile <= 25
|   |   |   |   |   |   |   |   firstQuartile <= 19
|   |   |   |   |   |   |   |   |   num_mismatches/overlap_length <= 0.013762: F (63.16/7.16)
|   |   |   |   |   |   |   |   |   num_mismatches/overlap_length > 0.013762
|   |   |   |   |   |   |   |   |   |   secondQuartile <= 20: T (5.02)
|   |   |   |   |   |   |   |   |   |   secondQuartile > 20: F (2.0)
|   |   |   |   |   |   |   |   firstQuartile > 19
|   |   |   |   |   |   |   |   |   secondQuartile <= 23: T (6.36)
|   |   |   |   |   |   |   |   |   secondQuartile > 23: F (2.0)
|   |   |   |   |   |   |   thirdQuartile > 25: F (105.63/5.56)
|   |   |   |   |   firstQuartile > 27: T (13.21)
|   |   |   |   num_mismatches/overlap_length > 0.014184: F (564.67/13.65)
|   |   |   thirdQuartile > 31
|   |   |   |   comp_overlap <= 1.24
```

```
|   |   |   |   |   firstQuartile <= 25
|   |   |   |   |   |   thirdQuartile <= 47: F (1125.38/9.49)
|   |   |   |   |   |   thirdQuartile > 47
|   |   |   |   |   |   |   num_mismatches/overlap_length <= 0.008264
|   |   |   |   |   |   |   |   thirdQuartile <= 52
|   |   |   |   |   |   |   |   |   firstQuartile <= 14
|   |   |   |   |   |   |   |   |   |   num_mismatches/overlap_length <= 0.007067: F (2.02/0.02)
|   |   |   |   |   |   |   |   |   |   num_mismatches/overlap_length > 0.007067: T (2.02/0.01)
|   |   |   |   |   |   |   |   |   firstQuartile > 14: F (50.0)
|   |   |   |   |   |   |   |   thirdQuartile > 52
|   |   |   |   |   |   |   |   |   thirdQuartile <= 75
|   |   |   |   |   |   |   |   |   |   num_mismatches/overlap_length <= 0.004525: T (4.01)
|   |   |   |   |   |   |   |   |   |   num_mismatches/overlap_length > 0.004525
|   |   |   |   |   |   |   |   |   |   |   comp_overlap <= 0.46
|   |   |   |   |   |   |   |   |   |   |   |   firstQuartile <= 14: T (2.07/0.01)
|   |   |   |   |   |   |   |   |   |   |   |   firstQuartile > 14
|   |   |   |   |   |   |   |   |   |   |   |   |   comp_overlap <= -0.55: F (10.03/0.0)
|   |   |   |   |   |   |   |   |   |   |   |   |   comp_overlap > -0.55
|   |   |   |   |   |   |   |   |   |   |   |   |   |   num_mismatches/overlap_length <= 0.00813: F (9.04/1.0)
|   |   |   |   |   |   |   |   |   |   |   |   |   |   num_mismatches/overlap_length > 0.00813: T (2.0)
|   |   |   |   |   |   |   |   |   |   |   comp_overlap > 0.46: F (3.02/0.01)
|   |   |   |   |   |   |   |   |   thirdQuartile > 75: T (5.12)
|   |   |   |   |   |   |   num_mismatches/overlap_length > 0.008264: F (326.22/7.18)
|   |   |   |   |   firstQuartile > 25: F (1626.15/4.88)
|   |   |   |   comp_overlap > 1.24
|   |   |   |   |   thirdQuartile <= 43
|   |   |   |   |   |   firstQuartile <= 12: F (6.0/0.0)
|   |   |   |   |   |   firstQuartile > 12: T (8.03/0.02)
|   |   |   |   |   thirdQuartile > 43: F (20.15/0.01)
comp_overlap > 1.47
|   thirdQuartile <= 39
|   |   comp_overlap <= 2.85
|   |   |   num_mismatches/overlap_length <= 0.003788: T (2301.26/1.1)
|   |   |   num_mismatches/overlap_length > 0.003788
|   |   |   |   firstQuartile <= 13
|   |   |   |   |   secondQuartile <= 16
|   |   |   |   |   |   thirdQuartile <= 13
|   |   |   |   |   |   |   thirdQuartile <= 12: T (361.73/11.04)
|   |   |   |   |   |   |   thirdQuartile > 12
|   |   |   |   |   |   |   |   comp_overlap <= 2.48
|   |   |   |   |   |   |   |   |   firstQuartile <= 7: T (22.19/3.0)
|   |   |   |   |   |   |   |   |   firstQuartile > 7
|   |   |   |   |   |   |   |   |   |   secondQuartile <= 12
|   |   |   |   |   |   |   |   |   |   |   comp_overlap <= 2.43
|   |   |   |   |   |   |   |   |   |   |   |   firstQuartile <= 8
|   |   |   |   |   |   |   |   |   |   |   |   |   comp_overlap <= 2.07: F (2.02/0.02)
|   |   |   |   |   |   |   |   |   |   |   |   |   comp_overlap > 2.07: T (5.04/1.0)
|   |   |   |   |   |   |   |   |   |   |   |   firstQuartile > 8
|   |   |   |   |   |   |   |   |   |   |   |   |   comp_overlap <= 2.22: T (4.11/0.0)
|   |   |   |   |   |   |   |   |   |   |   |   |   comp_overlap > 2.22: F (4.11/0.1)
|   |   |   |   |   |   |   |   |   |   |   comp_overlap > 2.43: F (5.09/0.09)
|   |   |   |   |   |   |   |   |   |   secondQuartile > 12: F (14.11/1.11)
|   |   |   |   |   |   |   |   comp_overlap > 2.48: T (52.63/4.01)
|   |   |   |   |   |   thirdQuartile > 13: T (227.96/3.17)
|   |   |   |   |   secondQuartile > 16
|   |   |   |   |   |   secondQuartile <= 20
|   |   |   |   |   |   |   comp_overlap <= 1.75: F (5.11/1.11)
|   |   |   |   |   |   |   comp_overlap > 1.75: T (14.32/2.02)
|   |   |   |   |   |   secondQuartile > 20: F (7.31/0.13)
|   |   |   |   firstQuartile > 13: T (117.11/0.23)
|   |   comp_overlap > 2.85
|   |   |   num_mismatches/overlap_length <= 0.01626
```

```
|   |   |   |   thirdQuartile <= 29: T (927853.46/17.81)
|   |   |   |   thirdQuartile > 29
|   |   |   |   |   thirdQuartile <= 38: T (30771.17/22.71)
|   |   |   |   |   thirdQuartile > 38
|   |   |   |   |   |   num_mismatches/overlap_length <= 0.007905: T (2569.78/10.86)
|   |   |   |   |   |   num_mismatches/overlap_length > 0.007905
|   |   |   |   |   |   |   firstQuartile <= 30
|   |   |   |   |   |   |   |   firstQuartile <= 25
|   |   |   |   |   |   |   |   |   firstQuartile <= 16
|   |   |   |   |   |   |   |   |   |   firstQuartile <= 14: T (14.96/1.98)
|   |   |   |   |   |   |   |   |   |   firstQuartile > 14: F (5.93)
|   |   |   |   |   |   |   |   |   firstQuartile > 16: T (20.0)
|   |   |   |   |   |   |   |   firstQuartile > 25: F (9.88)
|   |   |   |   |   |   |   firstQuartile > 30: T (120.0)
|   |   |   num_mismatches/overlap_length > 0.01626
|   |   |   |   thirdQuartile <= 26
|   |   |   |   |   secondQuartile <= 2
|   |   |   |   |   |   comp_overlap <= 4.03
|   |   |   |   |   |   |   num_mismatches/overlap_length <= 0.044025
|   |   |   |   |   |   |   |   secondQuartile <= 1
|   |   |   |   |   |   |   |   |   num_mismatches/overlap_length <= 0.018265: F (2.0)
|   |   |   |   |   |   |   |   |   num_mismatches/overlap_length > 0.018265: T (2.1/0.1)
|   |   |   |   |   |   |   |   secondQuartile > 1: T (14.72/0.1)
|   |   |   |   |   |   |   num_mismatches/overlap_length > 0.044025: F (4.0)
|   |   |   |   |   |   comp_overlap > 4.03: T (197.08/1.77)
|   |   |   |   |   secondQuartile > 2
|   |   |   |   |   |   num_mismatches/overlap_length <= 0.037634: T (37909.1/40.54)
|   |   |   |   |   |   num_mismatches/overlap_length > 0.037634
|   |   |   |   |   |   |   thirdQuartile <= 12: T (1107.6/3.95)
|   |   |   |   |   |   |   thirdQuartile > 12
|   |   |   |   |   |   |   |   secondQuartile <= 10
|   |   |   |   |   |   |   |   |   thirdQuartile <= 15
|   |   |   |   |   |   |   |   |   |   firstQuartile <= 8: T (64.98/0.99)
|   |   |   |   |   |   |   |   |   |   firstQuartile > 8
|   |   |   |   |   |   |   |   |   |   |   secondQuartile <= 9: F (6.91)
|   |   |   |   |   |   |   |   |   |   |   secondQuartile > 9: T (10.0)
|   |   |   |   |   |   |   |   |   thirdQuartile > 15
|   |   |   |   |   |   |   |   |   |   thirdQuartile <= 16
|   |   |   |   |   |   |   |   |   |   |   secondQuartile <= 8
|   |   |   |   |   |   |   |   |   |   |   |   firstQuartile <= 4: F (3.96/1.0)
|   |   |   |   |   |   |   |   |   |   |   |   firstQuartile > 4: T (2.0)
|   |   |   |   |   |   |   |   |   |   |   secondQuartile > 8: F (12.86/2.0)
|   |   |   |   |   |   |   |   |   |   thirdQuartile > 16
|   |   |   |   |   |   |   |   |   |   |   thirdQuartile <= 23: T (16.98)
|   |   |   |   |   |   |   |   |   |   |   thirdQuartile > 23: F (2.96/0.99)
|   |   |   |   |   |   |   |   secondQuartile > 10: T (972.69/7.91)
|   |   |   |   thirdQuartile > 26
|   |   |   |   |   firstQuartile <= 14
|   |   |   |   |   |   num_mismatches/overlap_length <= 0.02907
|   |   |   |   |   |   |   firstQuartile <= 7
|   |   |   |   |   |   |   |   thirdQuartile <= 29
|   |   |   |   |   |   |   |   |   firstQuartile <= 4: T (11.99/0.99)
|   |   |   |   |   |   |   |   |   firstQuartile > 4
|   |   |   |   |   |   |   |   |   |   secondQuartile <= 20
|   |   |   |   |   |   |   |   |   |   |   num_mismatches/overlap_length <= 0.023715: T (2.0)
|   |   |   |   |   |   |   |   |   |   |   num_mismatches/overlap_length > 0.023715: F (2.98/1.0)
|   |   |   |   |   |   |   |   |   |   secondQuartile > 20: F (9.88)
|   |   |   |   |   |   |   |   thirdQuartile > 29: T (17.0)
|   |   |   |   |   |   |   firstQuartile > 7: T (334.5/36.54)
|   |   |   |   |   |   num_mismatches/overlap_length > 0.02907
|   |   |   |   |   |   |   firstQuartile <= 13
|   |   |   |   |   |   |   |   secondQuartile <= 12: T (3.99)
```

```
|   |   |   |   |   |   |   |   secondQuartile > 12: F (30.7/7.99)
|   |   |   |   |   |   |   firstQuartile > 13: T (26.95/3.95)
|   |   |   |   |   firstQuartile > 14: T (1244.55/24.69)
|   thirdQuartile > 39
|   |   thirdQuartile <= 82
|   |   |   num_mismatches/overlap_length <= 0.005587
|   |   |   |   num_mismatches/overlap_length <= 0.004739
|   |   |   |   |   firstQuartile <= 35
|   |   |   |   |   |   secondQuartile <= 44: T (2458.29/38.64)
|   |   |   |   |   |   secondQuartile > 44
|   |   |   |   |   |   |   secondQuartile <= 55
|   |   |   |   |   |   |   |   firstQuartile <= 20: T (121.74/0.99)
|   |   |   |   |   |   |   |   firstQuartile > 20
|   |   |   |   |   |   |   |   |   thirdQuartile <= 54
|   |   |   |   |   |   |   |   |   |   firstQuartile <= 21
|   |   |   |   |   |   |   |   |   |   |   secondQuartile <= 46
|   |   |   |   |   |   |   |   |   |   |   |   thirdQuartile <= 48: T (6.99/0.99)
|   |   |   |   |   |   |   |   |   |   |   |   thirdQuartile > 48: F (3.96)
|   |   |   |   |   |   |   |   |   |   |   secondQuartile > 46: T (3.98)
|   |   |   |   |   |   |   |   |   |   firstQuartile > 21: T (77.91/5.94)
|   |   |   |   |   |   |   |   |   thirdQuartile > 54
|   |   |   |   |   |   |   |   |   |   firstQuartile <= 25
|   |   |   |   |   |   |   |   |   |   |   secondQuartile <= 52: F (6.93)
|   |   |   |   |   |   |   |   |   |   |   secondQuartile > 52: T (2.97/0.99)
|   |   |   |   |   |   |   |   |   |   firstQuartile > 25
|   |   |   |   |   |   |   |   |   |   |   firstQuartile <= 29: T (10.97)
|   |   |   |   |   |   |   |   |   |   |   firstQuartile > 29
|   |   |   |   |   |   |   |   |   |   |   |   thirdQuartile <= 58: F (7.93/1.99)
|   |   |   |   |   |   |   |   |   |   |   |   thirdQuartile > 58: T (2.99)
|   |   |   |   |   |   |   secondQuartile > 55: T (207.97/0.99)
|   |   |   |   |   firstQuartile > 35
|   |   |   |   |   |   firstQuartile <= 54
|   |   |   |   |   |   |   thirdQuartile <= 52: T (9388.75/13.87)
|   |   |   |   |   |   |   thirdQuartile > 52
|   |   |   |   |   |   |   |   firstQuartile <= 50
|   |   |   |   |   |   |   |   |   thirdQuartile <= 53: T (798.78/20.8)
|   |   |   |   |   |   |   |   |   thirdQuartile > 53
|   |   |   |   |   |   |   |   |   |   thirdQuartile <= 55
|   |   |   |   |   |   |   |   |   |   |   thirdQuartile <= 54
|   |   |   |   |   |   |   |   |   |   |   |   secondQuartile <= 52
|   |   |   |   |   |   |   |   |   |   |   |   |   num_mismatches/overlap_length <= 0
|   |   |   |   |   |   |   |   |   |   |   |   |   |   secondQuartile <= 44: T (2.0)
|   |   |   |   |   |   |   |   |   |   |   |   |   |   secondQuartile > 44: F (17.85/2.0)
|   |   |   |   |   |   |   |   |   |   |   |   |   num_mismatches/overlap_length > 0: T (2.97)
|   |   |   |   |   |   |   |   |   |   |   |   secondQuartile > 52: T (34.0)
|   |   |   |   |   |   |   |   |   |   |   thirdQuartile > 54: F (19.81)
|   |   |   |   |   |   |   |   |   |   thirdQuartile > 55: T (120.94/5.94)
|   |   |   |   |   |   |   |   firstQuartile > 50
|   |   |   |   |   |   |   |   |   thirdQuartile <= 56: T (2798.0)
|   |   |   |   |   |   |   |   |   thirdQuartile > 56
|   |   |   |   |   |   |   |   |   |   thirdQuartile <= 59: T (148.97/2.97)
|   |   |   |   |   |   |   |   |   |   thirdQuartile > 59
|   |   |   |   |   |   |   |   |   |   |   firstQuartile <= 53: T (3.98/0.99)
|   |   |   |   |   |   |   |   |   |   |   firstQuartile > 53: F (5.94)
|   |   |   |   |   |   firstQuartile > 54: T (4495.0)
|   |   |   |   num_mismatches/overlap_length > 0.004739
|   |   |   |   |   secondQuartile <= 52
|   |   |   |   |   |   secondQuartile <= 49: T (225.87/3.96)
|   |   |   |   |   |   secondQuartile > 49: F (22.79/0.99)
|   |   |   |   |   secondQuartile > 52: T (242.0)
|   |   |   num_mismatches/overlap_length > 0.005587
|   |   |   |   num_mismatches/overlap_length <= 0.016064
```

```
|  |  |  |  |  firstQuartile <= 35
|  |  |  |  |  |  secondQuartile <= 49
|  |  |  |  |  |  |  firstQuartile <= 33
|  |  |  |  |  |  |  |  secondQuartile <= 44
|  |  |  |  |  |  |  |  |  secondQuartile <= 16: T (37.93)
|  |  |  |  |  |  |  |  |  secondQuartile > 16
|  |  |  |  |  |  |  |  |  |  firstQuartile <= 20
|  |  |  |  |  |  |  |  |  |  |  firstQuartile <= 19
|  |  |  |  |  |  |  |  |  |  |  |  secondQuartile <= 32
|  |  |  |  |  |  |  |  |  |  |  |  |  firstQuartile <= 10: T (18.99)
|  |  |  |  |  |  |  |  |  |  |  |  |  firstQuartile > 10
|  |  |  |  |  |  |  |  |  |  |  |  |  |  num_mismatches/overlap_length <= 0.008889: T (15.91)
|  |  |  |  |  |  |  |  |  |  |  |  |  |  num_mismatches/overlap_length > 0.008889
|  |  |  |  |  |  |  |  |  |  |  |  |  |  |  firstQuartile <= 18
|  |  |  |  |  |  |  |  |  |  |  |  |  |  |  |  secondQuartile <= 24: F (4.95)
|  |  |  |  |  |  |  |  |  |  |  |  |  |  |  |  secondQuartile > 24: T (7.97/2.97)
|  |  |  |  |  |  |  |  |  |  |  |  |  |  |  firstQuartile > 18: T (3.0)
|  |  |  |  |  |  |  |  |  |  |  |  secondQuartile > 32
|  |  |  |  |  |  |  |  |  |  |  |  |  thirdQuartile <= 49
|  |  |  |  |  |  |  |  |  |  |  |  |  |  thirdQuartile <= 44
|  |  |  |  |  |  |  |  |  |  |  |  |  |  |  secondQuartile <= 37
|  |  |  |  |  |  |  |  |  |  |  |  |  |  |  |  thirdQuartile <= 42
|  |  |  |  |  |  |  |  |  |  |  |  |  |  |  |  |  firstQuartile <= 18: F (31.81/12.0)
|  |  |  |  |  |  |  |  |  |  |  |  |  |  |  |  |  firstQuartile > 18: T (8.98/1.98)
|  |  |  |  |  |  |  |  |  |  |  |  |  |  |  |  thirdQuartile > 42
|  |  |  |  |  |  |  |  |  |  |  |  |  |  |  |  |  secondQuartile <= 35: F (2.98/1.0)
|  |  |  |  |  |  |  |  |  |  |  |  |  |  |  |  |  secondQuartile > 35: T (11.0)
|  |  |  |  |  |  |  |  |  |  |  |  |  |  |  secondQuartile > 37
|  |  |  |  |  |  |  |  |  |  |  |  |  |  |  |  thirdQuartile <= 42: T (23.99/0.99)
|  |  |  |  |  |  |  |  |  |  |  |  |  |  |  |  thirdQuartile > 42: F (3.96)
|  |  |  |  |  |  |  |  |  |  |  |  |  |  thirdQuartile > 44: F (7.93/1.0)
|  |  |  |  |  |  |  |  |  |  |  |  |  thirdQuartile > 49: T (10.96/0.99)
|  |  |  |  |  |  |  |  |  |  |  firstQuartile > 19: T (48.0)
|  |  |  |  |  |  |  |  |  |  firstQuartile > 20
|  |  |  |  |  |  |  |  |  |  |  comp_overlap <= 3.99: F (10.71/1.52)
|  |  |  |  |  |  |  |  |  |  |  comp_overlap > 3.99
|  |  |  |  |  |  |  |  |  |  |  |  secondQuartile <= 27: F (11.19)
|  |  |  |  |  |  |  |  |  |  |  |  secondQuartile > 27
|  |  |  |  |  |  |  |  |  |  |  |  |  thirdQuartile <= 46
|  |  |  |  |  |  |  |  |  |  |  |  |  |  thirdQuartile <= 42
|  |  |  |  |  |  |  |  |  |  |  |  |  |  |  secondQuartile <= 35: F (6.53)
|  |  |  |  |  |  |  |  |  |  |  |  |  |  |  secondQuartile > 35
|  |  |  |  |  |  |  |  |  |  |  |  |  |  |  |  firstQuartile <= 25: T (13.0)
|  |  |  |  |  |  |  |  |  |  |  |  |  |  |  |  firstQuartile > 25
|  |  |  |  |  |  |  |  |  |  |  |  |  |  |  |  |  firstQuartile <= 30
|  |  |  |  |  |  |  |  |  |  |  |  |  |  |  |  |  |  secondQuartile <= 38: F (8.39)
|  |  |  |  |  |  |  |  |  |  |  |  |  |  |  |  |  |  secondQuartile > 38: T (2.93/0.93)
|  |  |  |  |  |  |  |  |  |  |  |  |  |  |  |  |  firstQuartile > 30
|  |  |  |  |  |  |  |  |  |  |  |  |  |  |  |  |  |  num_mismatches/overlap_length <= 0.007143: F (3.73)
|  |  |  |  |  |  |  |  |  |  |  |  |  |  |  |  |  |  num_mismatches/overlap_length > 0.007143: T (35.53/6.53)
|  |  |  |  |  |  |  |  |  |  |  |  |  |  thirdQuartile > 42
|  |  |  |  |  |  |  |  |  |  |  |  |  |  |  secondQuartile <= 41: F (30.77)
|  |  |  |  |  |  |  |  |  |  |  |  |  |  |  secondQuartile > 41
|  |  |  |  |  |  |  |  |  |  |  |  |  |  |  |  thirdQuartile <= 43: T (8.0)
|  |  |  |  |  |  |  |  |  |  |  |  |  |  |  |  thirdQuartile > 43: F (5.66/1.0)
|  |  |  |  |  |  |  |  |  |  |  |  |  thirdQuartile > 46: T (45.65/10.26)
|  |  |  |  |  |  |  |  secondQuartile > 44
|  |  |  |  |  |  |  |  |  thirdQuartile <= 50: T (120.97/0.99)
|  |  |  |  |  |  |  |  |  thirdQuartile > 50: F (20.84/5.98)
|  |  |  |  |  |  |  firstQuartile > 33: T (51.0)
|  |  |  |  |  |  secondQuartile > 49
|  |  |  |  |  |  |  thirdQuartile <= 58
```

```
|  |  |  |  |  |  |  |  firstQuartile <= 27
|  |  |  |  |  |  |  |  |  firstQuartile <= 26
|  |  |  |  |  |  |  |  |  |  thirdQuartile <= 54
|  |  |  |  |  |  |  |  |  |  |  secondQuartile <= 50: F (23.78/0.99)
|  |  |  |  |  |  |  |  |  |  |  secondQuartile > 50: T (12.0)
|  |  |  |  |  |  |  |  |  |  thirdQuartile > 54: F (24.78/1.99)
|  |  |  |  |  |  |  |  |  firstQuartile > 26: T (10.98/1.98)
|  |  |  |  |  |  |  |  firstQuartile > 27: F (56.47)
|  |  |  |  |  |  |  thirdQuartile > 58
|  |  |  |  |  |  |  |  num_mismatches/overlap_length <= 0.0125: T (60.97/0.99)
|  |  |  |  |  |  |  |  num_mismatches/overlap_length > 0.0125
|  |  |  |  |  |  |  |  |  secondQuartile <= 62: F (10.9/0.99)
|  |  |  |  |  |  |  |  |  secondQuartile > 62
|  |  |  |  |  |  |  |  |  |  thirdQuartile <= 73: T (5.0)
|  |  |  |  |  |  |  |  |  |  thirdQuartile > 73: F (2.97)
|  |  |  |  |  firstQuartile > 35
|  |  |  |  |  |  secondQuartile <= 44: T (605.98/1.98)
|  |  |  |  |  |  secondQuartile > 44
|  |  |  |  |  |  |  firstQuartile <= 52
|  |  |  |  |  |  |  |  thirdQuartile <= 53
|  |  |  |  |  |  |  |  |  num_mismatches/overlap_length <= 0.009217: T (253.97/2.97)
|  |  |  |  |  |  |  |  |  num_mismatches/overlap_length > 0.009217
|  |  |  |  |  |  |  |  |  |  firstQuartile <= 47
|  |  |  |  |  |  |  |  |  |  |  thirdQuartile <= 50
|  |  |  |  |  |  |  |  |  |  |  |  thirdQuartile <= 48
|  |  |  |  |  |  |  |  |  |  |  |  |  num_mismatches/overlap_length <= 0.00995: T (10.0)
|  |  |  |  |  |  |  |  |  |  |  |  |  num_mismatches/overlap_length > 0.00995: F (8.93/2.0)
|  |  |  |  |  |  |  |  |  |  |  |  thirdQuartile > 48: T (26.0)
|  |  |  |  |  |  |  |  |  |  |  thirdQuartile > 50
|  |  |  |  |  |  |  |  |  |  |  |  secondQuartile <= 50: F (13.87)
|  |  |  |  |  |  |  |  |  |  |  |  secondQuartile > 50: T (4.0)
|  |  |  |  |  |  |  |  |  |  firstQuartile > 47: T (77.99/0.99)
|  |  |  |  |  |  |  |  thirdQuartile > 53
|  |  |  |  |  |  |  |  |  thirdQuartile <= 55: F (102.06/2.0)
|  |  |  |  |  |  |  |  |  thirdQuartile > 55
|  |  |  |  |  |  |  |  |  |  thirdQuartile <= 73
|  |  |  |  |  |  |  |  |  |  |  num_mismatches/overlap_length <= 0.00995
|  |  |  |  |  |  |  |  |  |  |  |  thirdQuartile <= 62
|  |  |  |  |  |  |  |  |  |  |  |  |  thirdQuartile <= 58: T (14.99/0.99)
|  |  |  |  |  |  |  |  |  |  |  |  |  thirdQuartile > 58: F (2.97)
|  |  |  |  |  |  |  |  |  |  |  |  thirdQuartile > 62: T (41.0)
|  |  |  |  |  |  |  |  |  |  |  num_mismatches/overlap_length > 0.00995
|  |  |  |  |  |  |  |  |  |  |  |  thirdQuartile <= 65: F (17.85/2.0)
|  |  |  |  |  |  |  |  |  |  |  |  thirdQuartile > 65: T (5.0)
|  |  |  |  |  |  |  |  |  |  thirdQuartile > 73: F (11.91)
|  |  |  |  |  |  |  firstQuartile > 52: T (544.96/2.97)
|  |  |  |  num_mismatches/overlap_length > 0.016064
|  |  |  |  |  num_mismatches/overlap_length <= 0.035088
|  |  |  |  |  |  thirdQuartile <= 50
|  |  |  |  |  |  |  secondQuartile <= 46
|  |  |  |  |  |  |  |  thirdQuartile <= 42
|  |  |  |  |  |  |  |  |  firstQuartile <= 16
|  |  |  |  |  |  |  |  |  |  firstQuartile <= 9
|  |  |  |  |  |  |  |  |  |  |  firstQuartile <= 3: F (2.97)
|  |  |  |  |  |  |  |  |  |  |  firstQuartile > 3: T (9.0)
|  |  |  |  |  |  |  |  |  |  firstQuartile > 9: F (29.73/1.0)
|  |  |  |  |  |  |  |  |  firstQuartile > 16
|  |  |  |  |  |  |  |  |  |  secondQuartile <= 37
|  |  |  |  |  |  |  |  |  |  |  num_mismatches/overlap_length <= 0.024096: T (54.95/4.95)
|  |  |  |  |  |  |  |  |  |  |  num_mismatches/overlap_length > 0.024096: F (8.92)
|  |  |  |  |  |  |  |  |  |  secondQuartile > 37: T (64.0)
|  |  |  |  |  |  |  |  thirdQuartile > 42
```

```
|  |  |  |  |  |  |  |  |  secondQuartile <= 27
|  |  |  |  |  |  |  |  |  |  num_mismatches/overlap_length <= 0.028: T (33.99)
|  |  |  |  |  |  |  |  |  |  num_mismatches/overlap_length > 0.028
|  |  |  |  |  |  |  |  |  |  |  secondQuartile <= 21: F (2.97)
|  |  |  |  |  |  |  |  |  |  |  secondQuartile > 21: T (2.0)
|  |  |  |  |  |  |  |  |  secondQuartile > 27
|  |  |  |  |  |  |  |  |  |  firstQuartile <= 39
|  |  |  |  |  |  |  |  |  |  |  secondQuartile <= 44
|  |  |  |  |  |  |  |  |  |  |  |  thirdQuartile <= 43
|  |  |  |  |  |  |  |  |  |  |  |  |  secondQuartile <= 39: F (48.55/1.0)
|  |  |  |  |  |  |  |  |  |  |  |  |  secondQuartile > 39: T (3.0)
|  |  |  |  |  |  |  |  |  |  |  |  thirdQuartile > 43: F (69.36/1.0)
|  |  |  |  |  |  |  |  |  |  |  secondQuartile > 44
|  |  |  |  |  |  |  |  |  |  |  |  firstQuartile <= 29: T (7.98)
|  |  |  |  |  |  |  |  |  |  |  |  firstQuartile > 29: F (8.92)
|  |  |  |  |  |  |  |  |  |  firstQuartile > 39
|  |  |  |  |  |  |  |  |  |  |  thirdQuartile <= 47: T (13.0)
|  |  |  |  |  |  |  |  |  |  |  thirdQuartile > 47: F (4.96/1.0)
|  |  |  |  |  |  |  secondQuartile > 46
|  |  |  |  |  |  |  |  num_mismatches/overlap_length <= 0.028169
|  |  |  |  |  |  |  |  |  firstQuartile <= 37: T (46.98/1.98)
|  |  |  |  |  |  |  |  |  firstQuartile > 37
|  |  |  |  |  |  |  |  |  |  firstQuartile <= 44: F (3.96)
|  |  |  |  |  |  |  |  |  |  firstQuartile > 44: T (2.0)
|  |  |  |  |  |  |  |  num_mismatches/overlap_length > 0.028169: T (63.0)
|  |  |  |  |  |  thirdQuartile > 50
|  |  |  |  |  |  |  firstQuartile <= 47
|  |  |  |  |  |  |  |  secondQuartile <= 32
|  |  |  |  |  |  |  |  |  firstQuartile <= 18: T (6.99)
|  |  |  |  |  |  |  |  |  firstQuartile > 18: F (2.0)
|  |  |  |  |  |  |  |  secondQuartile > 32: F (167.63/13.98)
|  |  |  |  |  |  |  firstQuartile > 47
|  |  |  |  |  |  |  |  thirdQuartile <= 59
|  |  |  |  |  |  |  |  |  num_mismatches/overlap_length <= 0.017391: F (6.93)
|  |  |  |  |  |  |  |  |  num_mismatches/overlap_length > 0.017391: T (38.99/0.99)
|  |  |  |  |  |  |  |  thirdQuartile > 59
|  |  |  |  |  |  |  |  |  thirdQuartile <= 64: F (15.85)
|  |  |  |  |  |  |  |  |  thirdQuartile > 64: T (54.99/0.99)
|  |  |  |  |  num_mismatches/overlap_length > 0.035088
|  |  |  |  |  |  firstQuartile <= 32: F (112.96/3.0)
|  |  |  |  |  |  firstQuartile > 32
|  |  |  |  |  |  |  secondQuartile <= 39: T (5.0)
|  |  |  |  |  |  |  secondQuartile > 39: F (43.61/1.0)
|  |  thirdQuartile > 82
|  |  |  firstQuartile <= 27
|  |  |  |  secondQuartile <= 87: T (107.2/5.94)
|  |  |  |  secondQuartile > 87: F (6.93)
|  |  |  firstQuartile > 27
|  |  |  |  num_mismatches/overlap_length <= 0.008511
|  |  |  |  |  firstQuartile <= 71
|  |  |  |  |  |  num_mismatches/overlap_length <= 0.00578
|  |  |  |  |  |  |  secondQuartile <= 77
|  |  |  |  |  |  |  |  secondQuartile <= 67
|  |  |  |  |  |  |  |  |  num_mismatches/overlap_length <= 0.005291: T (7.93/0.99)
|  |  |  |  |  |  |  |  |  num_mismatches/overlap_length > 0.005291: F (5.94)
|  |  |  |  |  |  |  |  secondQuartile > 67: T (15.85)
|  |  |  |  |  |  |  secondQuartile > 77
|  |  |  |  |  |  |  |  thirdQuartile <= 89: F (3.96)
|  |  |  |  |  |  |  |  thirdQuartile > 89
|  |  |  |  |  |  |  |  |  firstQuartile <= 43: F (13.87/1.98)
|  |  |  |  |  |  |  |  |  firstQuartile > 43: T (12.88/4.95)
|  |  |  |  |  |  num_mismatches/overlap_length > 0.00578
```

```
|   |   |   |   |   |   |   |   secondQuartile <= 82: F (43.59/0.99)
|   |   |   |   |   |   |   |   secondQuartile > 82
|   |   |   |   |   |   |   |   |   firstQuartile <= 61: T (4.95)
|   |   |   |   |   |   |   |   |   firstQuartile > 61: F (3.96/0.99)
|   |   |   |   |   |   firstQuartile > 71: F (49.53/3.96)
|   |   |   |   |   num_mismatches/overlap_length > 0.008511: F (599.39/6.93)

Number of Leaves  : 	311

Size of the tree : 	621
```
